# Multi-sensor IoT data and AI-driven adaptive control model for library indoor environments

**DOI:** 10.1371/journal.pone.0361038

**Published:** 2026-10-27

**Authors:** Mengxi Zhang

**Affiliations:** Library, Shandong Jiaotong University, Jinan, Shandong, China; Universidad Tecnica Federico Santa Maria, CHILE

## Abstract

Modern library areas require constant environmental adjustment to preserve user comfort and promote learning efficiency. This investigation offers a Multi-Sensor Internet of Things (IoT) Data Source and Artificial Intelligence-Based Adaptation Control Model for Library Indoor Systems, which is intended to transform heterogeneous sensing data into intelligent real-time environmental management decisions. A Multi-Sensor IoT Library Indoor Environment Dataset containing 2,745 time-stamped observations was developed, thereby capturing dynamic indoor environmental variations. Normalization and noise smoothing processes were performed before the analysis of the collected data for improving the quality of the analysis. In order to detect the most useful features of the environment, Principal Component Analysis (PCA) was used to perform dimension reduction and simplify calculations. The proposed approach includes edge sensing, intelligent data processing, and actuator cooperation for implementing an efficient closed-loop environmental management system. To classify indoor environmental conditions, a Colony Predation Algorithm enhanced with Random Adaptive Boosting (CPA-RAdaBoost) was developed, where CPA maximizes feature weights and model hyperparameters, Random Forest (RF) enhances ensemble variety, and Adaptive Boosting strengthens the identification of complicated and border environmental conditions. The adaptive control instructions for HVAC, lighting and ventilation are formulated considering the prevailing environmental conditions to facilitate indoor environment control. Experimental implementation using Python demonstrates the effectiveness of the proposed framework, reaching a MAE of 3.9 and control accuracy of 97.1%, indicating its capacity for intelligent, responsive, and energy-efficient smart library environmental management.

## Introduction

The Indoor Environmental Quality (IEQ) is highly influential in terms of people’s health and well-being in terms of the occupied space, particularly in case of education-oriented buildings such as libraries, in which people spend lots of time reading and studying [1]. It has been noted that the well-maintained indoor environment with appropriate temperature, ventilation, lighting, and noise levels enhances efficiency of learning and study process as well as users’ satisfaction with the performance [2]. As buildings become more advanced through their construction and development, sensors integrated into IoT technology detect changes in the indoor environment providing real-time data and controlling buildings’ systems [3]. At the same time, AI appears to be among the most efficient tools for analysis and decision-making based on the gathered data on the environment [4]. Adaptive IEQ management is made possible through the use of IoT and AI technologies due to the ability to continually modify the building system based on changing conditions [5]. Adaptive Regulation of Intelligent IoT-Based Smart Library Environment is demonstrated in Fig 1.

**Fig 1:**
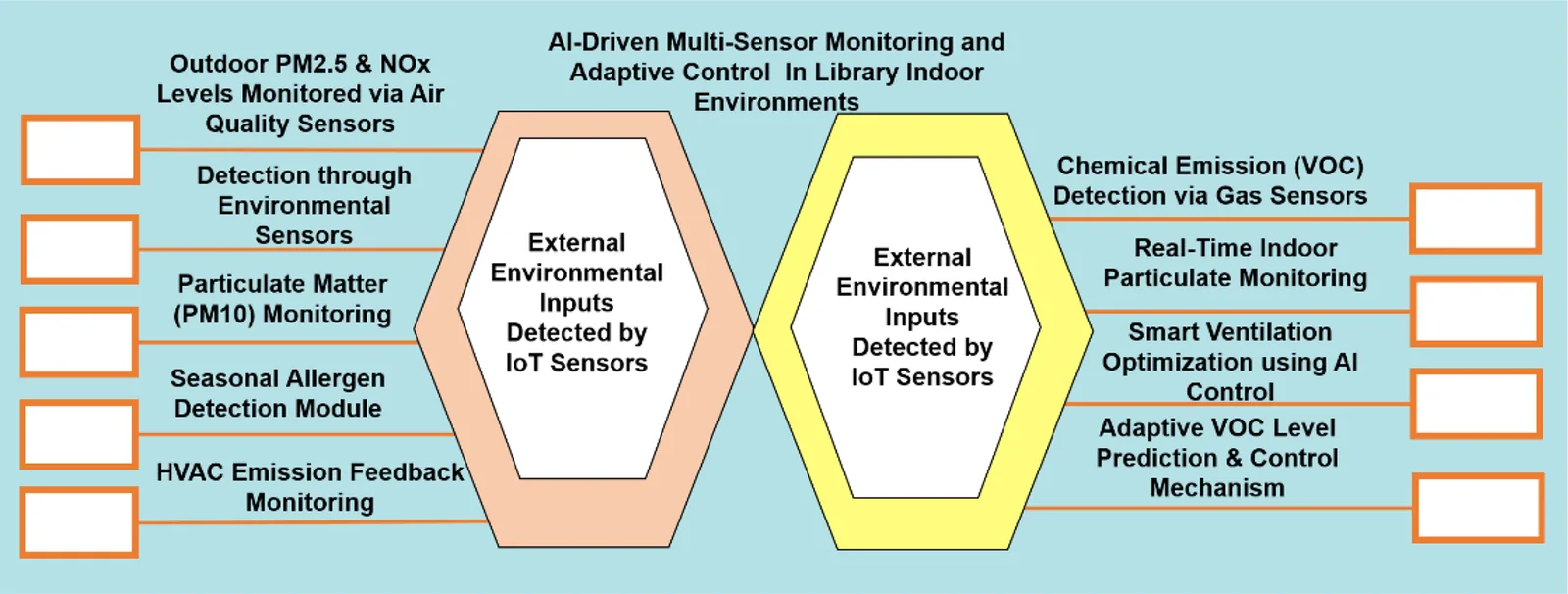
AI-based multi-sensors system for intelligent control of library indoor environment.

Recent research efforts in intelligent Heating, Ventilation, and Air Conditioning (HVAC) systems have concentrated on optimizing energy efficiency without compromising the comfort of the occupants. The use of machine learning (ML) methods to predict temperature preference, occupancy behavior, and energy demand in buildings using past and current data is becoming more popular [6]. The use of advanced AI algorithms such as deep learning algorithms, ensemble algorithms, and nature-inspired optimization algorithms helps in prediction and making intelligent decisions for controlling the indoor environment [7]. On the other hand, the rapid advancement in IoT technology has made it possible to deploy low-cost and connected sensing systems capable of collecting environmental data [8]. Whereas many recent research works have used several factors that pertain to the internal environment, such as temperature, humidity, CO_2_ level, lighting, occupancy, air quality, and noise, current systems generally use one or a few factors and not the entire combination, and most current systems focus mainly on the aspects of monitoring, prediction, or assessment of the indoor environment. While IoT-based solutions generally include real-time sensing and visualization, the machine learning models are generally used only for the purpose of prediction. In addition to that, very few research works have combined heterogeneous multi-sensor data, optimization of features, intelligent environmental state recognition, and actuation in a single solution for library environments. Therefore, a requirement arises for an adaptive control solution based on artificial intelligence [9,10].

## Related works

Traditional environmental management in indoor settings is usually achieved by controlling the system on the basis of preset rules or operation schedules. Traditional HVAC systems work based on static temperature settings, whereas lighting systems account for dynamic occupancy trends and environmental changes [11]. Traditional applications of environmental control have been observed to make use of PID controllers for HVAC systems. The problem with these controllers is that they need to be tuned properly and cannot perform well when operating under varied conditions, disturbances, and environmental uncertainties [12]. Therefore, traditional methods of environment management in libraries cannot offer adaptive control and optimal energy use in response to environmental changes during the day [13].

### Thermal comfort and IEQ assessment

Evaluating thermal comfort conditions using occupant surveys, feature reduction using PCA, and machine learning approaches was discussed in [14]. In this research, both physiological and perception-related responses were collected from library users under different indoor thermal conditions. PCA was utilized in order to improve feature selection based on the transformation of 61 input features into 43 principal components without losing more than 90% of the total variance of the data. A method for estimating students’ acoustic perceptions in academic library environments using machine learning was proposed in [15]. On-site acoustic measurements, environmental characteristics, and information about the occupant derived from surveys were used in the analysis. The RF and GBM algorithms showed better performance in terms of classification, achieving prediction accuracy equal to 0.87 and 0.84 for acoustic acceptance estimation, respectively.

The evaluation of IEQ in libraries was carried out using field monitoring and renovation approaches in [16]. The experiment used HOBO data logging devices. The data collected showed that the temperature in the rare collection room exceeded 21.2 °C, while the relative humidity was kept between 51.3–55.8%, CO2 was measured at 588 ppm, and acoustic values varied from 43–61 dB(A). Nevertheless, the use of the suggested renovation solutions. The research [17] investigated the thermal behavior and IAQ of library buildings by means of occupants’ surveys, on-site measurements, and CFD modeling. The results revealed the preferred thermal conditions of 26 °C for adults and 24 °C for kids with an air velocity of 0.2 m/s; moreover, the use of the Total Heat Exchanger (THX) system lowered the CO2 level up to 200 ppm.

### ML-based prediction and energy optimization

An advanced multi-objective optimization technique that incorporates machine learning to improve indoor environmental quality and building energy efficiency is proposed in [18]. The research involved analysis of 44 typical atrium designs of Wuhan University libraries. The optimal atrium design provided improvements of 29.46% in terms of energy saving, 10.46% in thermal comfort, and 65.56% regarding daylight usage, while ML models decreased the prediction time by almost 400 times. An ML prediction framework to control IAQ and energy balance of educational buildings was suggested in [19]. The research included 35,000 + environmental samples, including CO_2_, PM, temperature, humidity, and other factors like temporal and rainfall information. Prediction accuracy of GRU- and LSTM-based models reached above 92%.

The research paper [20] has come up with a Levenberg–Marquardt algorithm-based optimization process coupled with a global search mechanism for minimizing energy utilization by HVAC systems in library buildings. The method made use of real-life energy utilization data along with HVAC control variables collected from a university library building. Over 30% reduction in energy utilization was made possible without compromising the indoor thermal comfort of the users. A CNN-based occupant prediction model for HVAC control in a library building has been developed in [21]. This method relied upon real-time occupant details from the reading hall of Nanchang University library building in conjunction with HVAC data. The energy utilization was reduced by 52.1%, whereas the Predicted Percentage of Dissatisfied (PPD) index value was reduced from 14.3% to 7.2%.

Thermal conditions indoors and local climate variations in a Xinjiang library setting were assessed in [22] using questionnaire assessment results combined with computer simulation. Questionnaire responses provided by the library students and staff were correlated with field data of temperature, moisture, and airflow measurements. As a result, the research revealed a neutral PMV value of −0.11 to 0.81, and increased airflow windows were opened at a 60° angle. The thermal comfort of the library open space was investigated in [23] using CFD-based simulation of airflow in combination. Field tests have allowed measurement of temperature, relative humidity, and radiant temperature values at eight different points in the library. According to the simulation results, the temperature and humidity were more than 5.5% accurate, while PMV and TSV prediction errors did not exceed 12.2% and 2.7%, respectively.

An evaluation of thermal comfort analysis with respect to atria in library settings was conducted by [24] via field measurements and occupant surveys, based on TRNSYS-CONTAM simulation tools. The indoor temperature difference in the winter period was 4.7 °C, while occupant dissatisfaction was 27%, whereas the simulation-based technique resulted in decreasing the vertical temperature stratification by 1.5–2 °C. Thermal comfort enhancement and energy saving studies via air temperature and operative temperature zoning approaches were studied in [25]. Air and operative temperatures were measured in reading areas of the library, considering various directions of rooms and indoor zones. The adaptive temperature zoning approach resulted in a decrease in energy consumption, where the upper floor limit was decreased by 0.7 °C, and the northern zone was increased by 2.0 °C.

### IoT-based monitoring and smart libraries

A simulation-based analysis of low energy consumption cooling systems in library buildings located in hot-humid regions was carried out in [26]. This study involved the use of SET readings and energy consumption associated with cooling gathered from a library building in the city of Nanning. The installation of cooling shutters decreased the SET by 3.0 °C, and ceiling fans lowered it by 4.5 °C. The combined operation of both cooling measures achieved an overall decrease of 60.8% in cooling energy demand. An intelligent IAQ monitoring and prediction framework using deep learning and IoT sensors was proposed in [27]. Measurements of CO_2_, PM2.5, TVOCs, temperature, and humidity were made in real time in a university environment, whereby LSTM obtained high accuracy of IAQ predictions based on a temperature prediction MAE of 0.07. An adaptive IAQ assessment framework based on IoT sensors, Kalman filter, ANFIS, and Markov chains was developed in [28]. Though the forecasting error was 3.47%, poor IAQ states were found for most of the periods without including occupants’ feedback.

An IoT solution using ESP32 and MQTT communication for indoor environmental evaluation of laboratory, classroom, and library spaces with various occupancy was suggested in [29]. This system acquired real-time sensor data with a measurement error of 2%, a communication delay between 215 and 310 ms, a packet delivery ratio of 99.1%, and an R^2^ score over 0.98. However, this system had limitations in sensor data acquisition since it did not include sound, occupants’ behavior, and additional comfort factors. An intelligent system to optimize the library environment incorporating IoT sensing, machine learning, and a feedback mechanism was designed in [30]. Sensor data, occupancy detection data via images, survey results, and time-related data were gathered at university libraries. KNN provided the best accuracy with a 98.1% F1-score. But the use of subjective surveys can introduce bias into the process.

### Research gaps

While previous research has managed to enhance library indoor environmental management through IoT sensors, machine learning, thermal comfort, and energy management techniques, there are some shortcomings that need to be addressed. The majority of the methodologies are centered upon monitoring or predicting environmental parameters individually without developing an overall multi-parameter adaptive control methodology.

To fill this gap, this study offers a closed-loop framework for Multi-Sensor IoT-Enabled adaptive control using Principal Component Analysis and the CPA-RAdaBoost model. The proposed Multi-Sensor IoT-Enabled Adaptive Control framework combines multi-sensor data fusion, feature extraction, environmental state detection, and decision-making into a closed-loop design. Furthermore, conventional machine learning algorithms have little capability in optimizing feature importance, understanding the non-linear relationship between sensors, and performing real-time adaptive control for heating, ventilation, air conditioning, and lighting. This research aims to introduce a new Multi-Sensor IoT Data and AI-Driven Adaptive Control Model which combines Principal Component Analysis (PCA), Random Forest, AdaBoost, and Colony Predation Algorithm (CPA). While in previous research the models could perform one or two of those tasks, the introduced CPA-RAdaBoost algorithm can do all of them at once.

**Based on the identified research gap, this study addresses the following research questions:**

- **RQ1:** How can heterogeneous multi-sensor IoT data (temperature, humidity, CO_2_ concentration, illumination, noise, and occupancy) be effectively integrated to provide accurate and comprehensive real-time environmental state recognition in smart library environments?
- **RQ2:** Can the proposed CPA-RAdaBoost framework improve environmental state recognition by optimizing feature selection, learning nonlinear relationships among multi-sensor data, and accurately classifying dynamic indoor environmental conditions compared with conventional machine learning approaches?
- **RQ3:** What degree of effectiveness will the known states of the environment be able to achieve in order to transform into intelligent adaptive controls for HVAC, lighting, and ventilation systems?

**Proposed Framework Scope:** The presented CPA-RAdaBoost method is an artificial intelligence-based system that supports environmental state classification and adaptive control decisions in the library environment. The approach involves data collected from several sensors in IoT and allows classifying indoor environmental state and generating control signals for HVAC and lighting systems based on predetermined patterns without accounting for thermal inertia, dynamics, delay, and occupants’ behavior. The library has been selected as the object of investigation because of the necessity of strict control of indoor environment for comfortable studying. Nevertheless, the approach can be adjusted for any indoor environment.

Innovation of CPA-RAdaBoost algorithm is that these four techniques are combined together into one closed loop control system. By using PCA, redundancies in sensor data can be removed, and by using Random Forest, nonlinear relations in the environment can be captured, while by using AdaBoost, hard states in the environment can be learned, and by using CPA, best weight and hyperparameters are achieved. The combination of these techniques allows an immediate move from Recognition to Adaptive HVAC & Ventilation Control. Contributions of research are listed below.

- Unified multi-sensor framework for IoT applications is designed for integration of heterogeneous environmental information regarding indoor environments of the library.
- Proposed is an innovative method for feature selection and environmental state recognition using the CPARRAdaBoost algorithm which integrates Principal Component Analysis, Canonical Power Analysis, Random Forests, and Adaboost.
- Adaptive control system design in closed loop is intended to control HVAC, lighting, and ventilation through automatically recognizing environmental conditions.
- Experimental results demonstrate superior accuracy, lower prediction error, reduced response latency, and effective adaptive environmental control compared with existing methods.

### Problem statement

Although there have been considerable developments in IoT-based environmental sensing, machine learning predictions, and intelligent building management, the current state-of-the-art in library environmental management is still piecemeal and mostly concentrates on monitoring or predicting a single environmental parameter. There is no extensive literature that brings together multiple sensor data for adaptive control of heating, ventilation, and air conditioning, along with lights in response to dynamic occupancy conditions and the environment. This work addresses this need by using a framework that utilizes PCA for dimensionality reduction, Random Forests and AdaBoost for environmental state recognition, and CPA for feature weights and hyperparameter optimization.

**Research Motivation and Context:** Current indoor environmental control systems for libraries primarily concentrate on the surveillance or prediction of single environmental parameters with little ability to perform multiple sensor data analysis and adaptive control. Furthermore, many of these systems use a static approach or depend on the human perception of the environment, making them less relevant to dynamic library environments. In order to overcome these challenges, this research aims to develop an intelligent library environment management system based on Multi-Sensor Internet of Things and Artificial Intelligence for adaptive control, which uses PCA-based feature extraction combined with the CPA-RAdaBoost model for environmental state recognition.

## CPA-RAdaBoost-based environmental state recognition and feature prioritization

The proposed CPA-RAdaBoost method is an intelligence-learning-based approach that analyzes and manages various indoor environmental factors within library premises. The model adopts the use of RF as its base learner, with many DTs created from random selection of training instances and PCA-based features. The AdaBoost scheme is used in order to increase classification performance and accuracy of the ensemble model. This way, the intelligent approach can detect small changes in the environment, such as localized thermal discomfort and gradual changes in the air quality, that have an impact on the learning comfort of the occupants. CPA optimization is used to make the search process more efficient by balancing global and local search, minimizing convergence errors, and choosing important features such as CO2 levels, temperature changes, and occupancy trends. Fig 2 demonstrates the layer activation process within the proposed approach for intelligent indoor environmental optimization.

**Fig 2:**
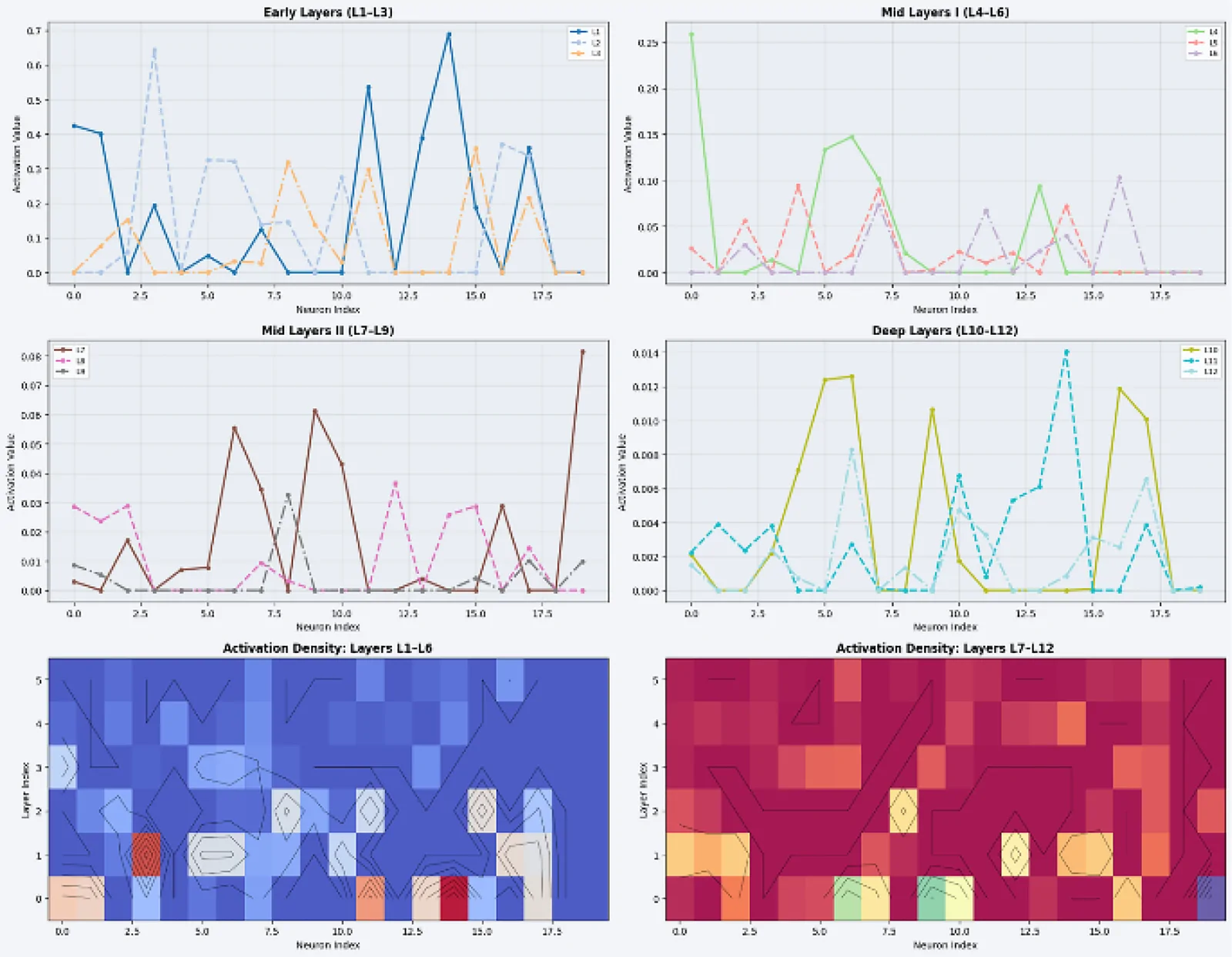
Layer-wise activation evaluation illustrating adaptive AI-guided environmental state identification in advanced smart library systems.

The new CPA-RAdaBoost model is based on the combination of CPA, Random Forest (RF), and AdaBoost to leverage the advantages of these components in order to build an efficient solution for intelligent environmental control in the library. RF is used as the basic classifier because it can handle the nonlinear relationship between heterogeneous IoT sensor data and has the ability to be resilient to noise. The use of AdaBoost allows us to achieve better classification results, as this technique emphasizes misclassified and difficult-to-classify environmental states through multiple iterations. CPA acts as the global optimization technique to optimize the features’ weights and some important hyperparameters of RF.

**Justification of the Proposed Framework:** PCA is used for minimizing redundancy and increasing efficiency in computation while retaining important information from the environment. Random Forest algorithm has been chosen due to its capability of modeling the nonlinear relations among heterogeneous sensor data collected by the Internet of Things with maximum robustness. AdaBoost can be used to classify data by placing greater importance on the difficult states of the environment, which in turn helps to increase the prediction accuracy. CPA optimizes itself automatically with regard to feature weights and hyperparameters, which leads to better convergence.

### Sensor data preprocessing for intelligent environmental regulation

The scaling using z-score is employed as pre-processing step on the heterogeneous data collected from the IoT sensors in the indoor library environment, ensuring consistent data distribution for parameters such as temperature, relative humidity, CO2 content, lighting, sound, and presence of people. Normalization of each sensor parameter, x, is illustrated through Equation (1).


$$\begin{array}{c} z=\frac{x-\mu }{\sigma } \end{array}$$ (1)

where μ is the mean value of the collected sensor readings and σ is their standard deviation. With this technique, the distribution of sensor data becomes closer to zero mean but with unit standard deviation, hence improving the reliability and precision of AI-based indoor environmental condition detection. Fig 3 shows the process of normalization of IoT sensor data for adaptive intelligent control of library environment.

**Fig 3:**
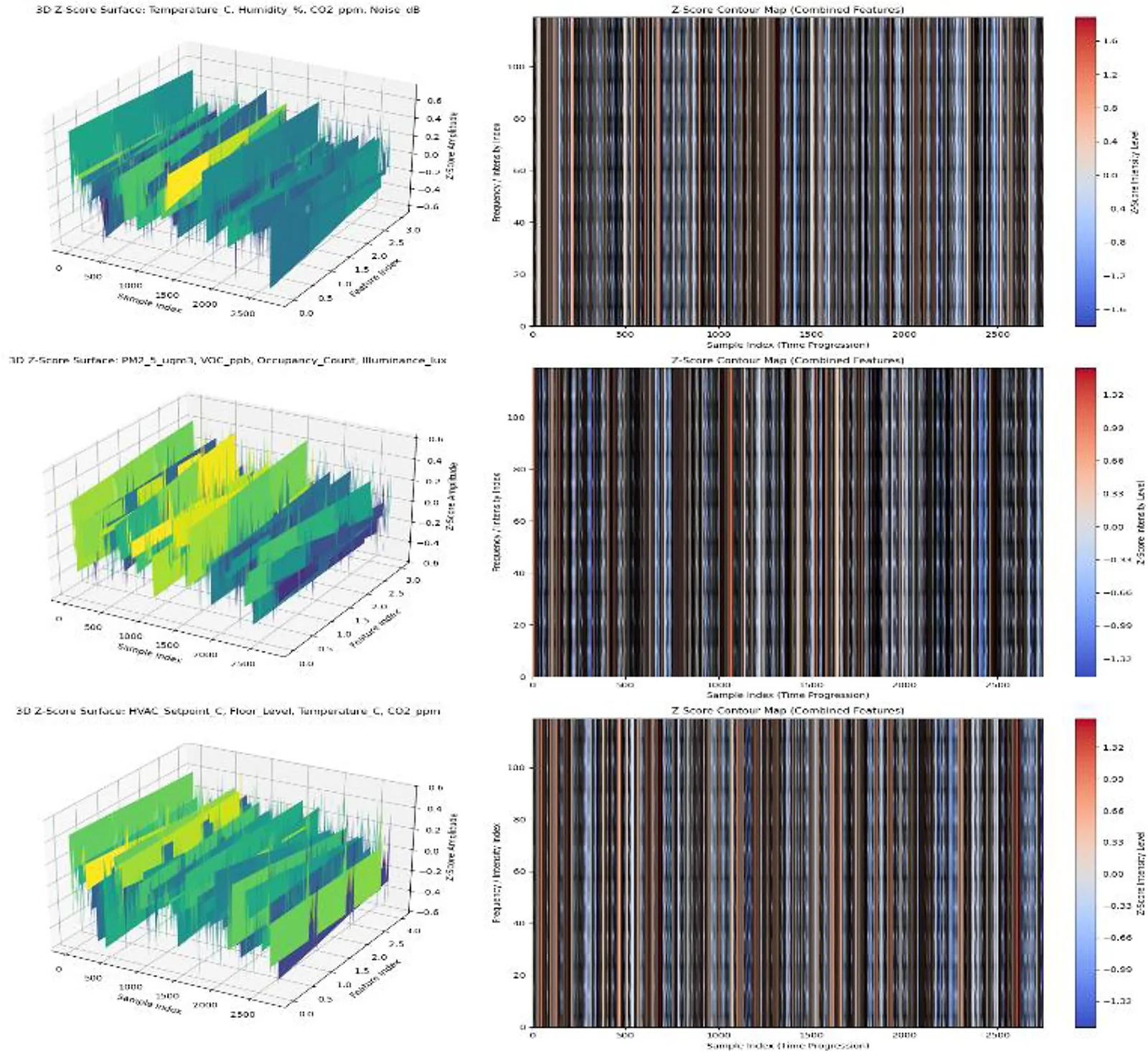
Original and normalized distribution of IoT features assessment for adaptive smart library environment control.

To improve the signal quality while preserving its temporal aspects, the Savitzky-Golay (SG) filter method is used. In SG filtering, noise is reduced by approximating the signal in a moving window based on a polynomial regression model of order m on a 2m + 1 signal samples window. Signal filtration is done according to the formula presented in Equation (2).


$$\begin{array}{c} \hat{x}(t)=\sum_{k=-m}^{m}c_{k}x(t+k) \end{array}$$ (2)

where $\hat{x}(t)$ is the filtered sensor signal at instant t, x(t+k) is the value of the sensor signal at adjacent instants within the selected window, and $c_{k}$ stands for the values of the convolution coefficients based on the process of polynomial approximation. This filtering technique retains the vital changes and trends of indoor environmental conditions while minimizing any unnecessary changes. Fig 4 demonstrates the enhanced quality of IoT signals following the SG filter for smart library management

**Fig 4:**
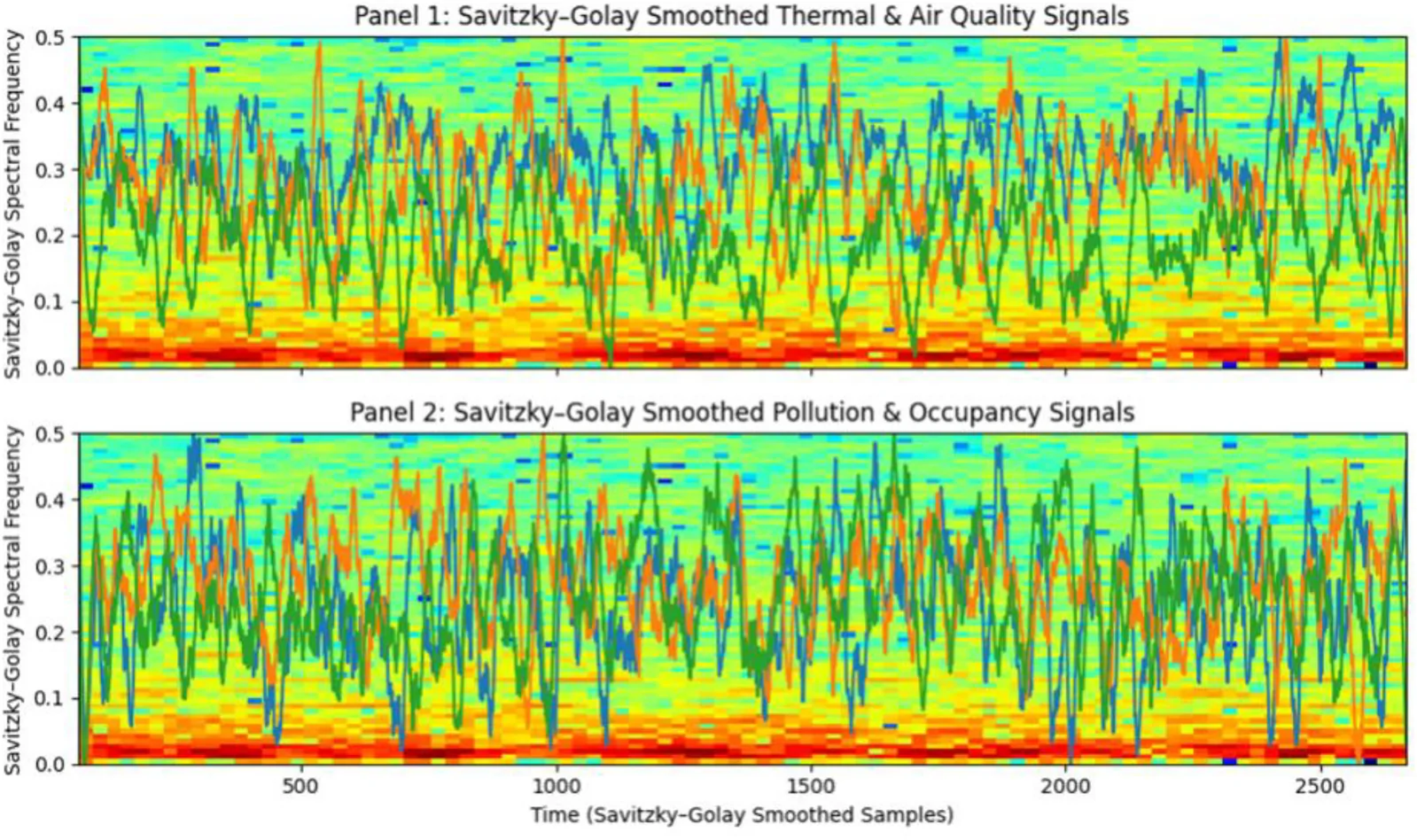
Environmental state predictions in smart libraries via SG-smoothed IoT signals.

### PCA-based feature compression and redundancy reduction

PCA is used for the transformation of the highly correlated variables such as temperature, humidity, carbon dioxide concentration, light level, sound level, and human density from the smart library sensor data streams into orthogonal components. The technique reduces the dimensionality by retaining the most influential variations of the indoor environment, reducing redundancy in information from the sensor features, and enabling efficient state identification and environmental control.

Covariance matrix S for the dataset $W_{l}$ obtained from the library data is determined using Equation (3), where the deviation of each of the observed values of the sensor from the mean vector is taken into account. The summing operation determines the effect of all the observed environmental parameters or features based on the IoT. PCA reduces the correlated sensor values (temperature, humidity, CO_2_, illumination, noise, and occupancy) to orthogonal compact components. Covariance matrix Dfor M observed values $W_{l}$ is determined using the mean vector $\overset{↼}{W}$ (Equations 3–4).


$$\begin{array}{c} D=\sum_{l=\text{1}}^{M}(W_{l}-\overset{↼}{W})(W_{l}-\overset{↼}{W}{)}^{S} \end{array}$$ (3)

Equation (4) represents the calculation of the mean value of the data points gathered, which is done by summing up all feature vectors from the sensor readings in the dataset. This procedure gives the baseline used in detecting environmental changes in the library setting.


$$\begin{array}{c} \overset{↼}{W}=\sum_{l=\text{1}}^{M}\frac{W_{l}}{M} \end{array}$$ (4)

$$\begin{array}{c} Du_{j}=\lambda_{j}u_{j} \end{array}$$ (5)

In Equation 5, D Eigen-Decomposition yields eigenvectors $u_{j}$ and eigenvalues $\lambda_{j}$, where the eigenvalue-based ranking is descending for the components. Fifteen of the components are selected to compose the new feature set $W_{NEW}$ (Equation 6). Fig 5 illustrates the feature interrelationships and environmental conditions.


$$\begin{array}{c} W_{NEW}=W_{u_{j}} \end{array}$$ (6)

**Fig 5:**
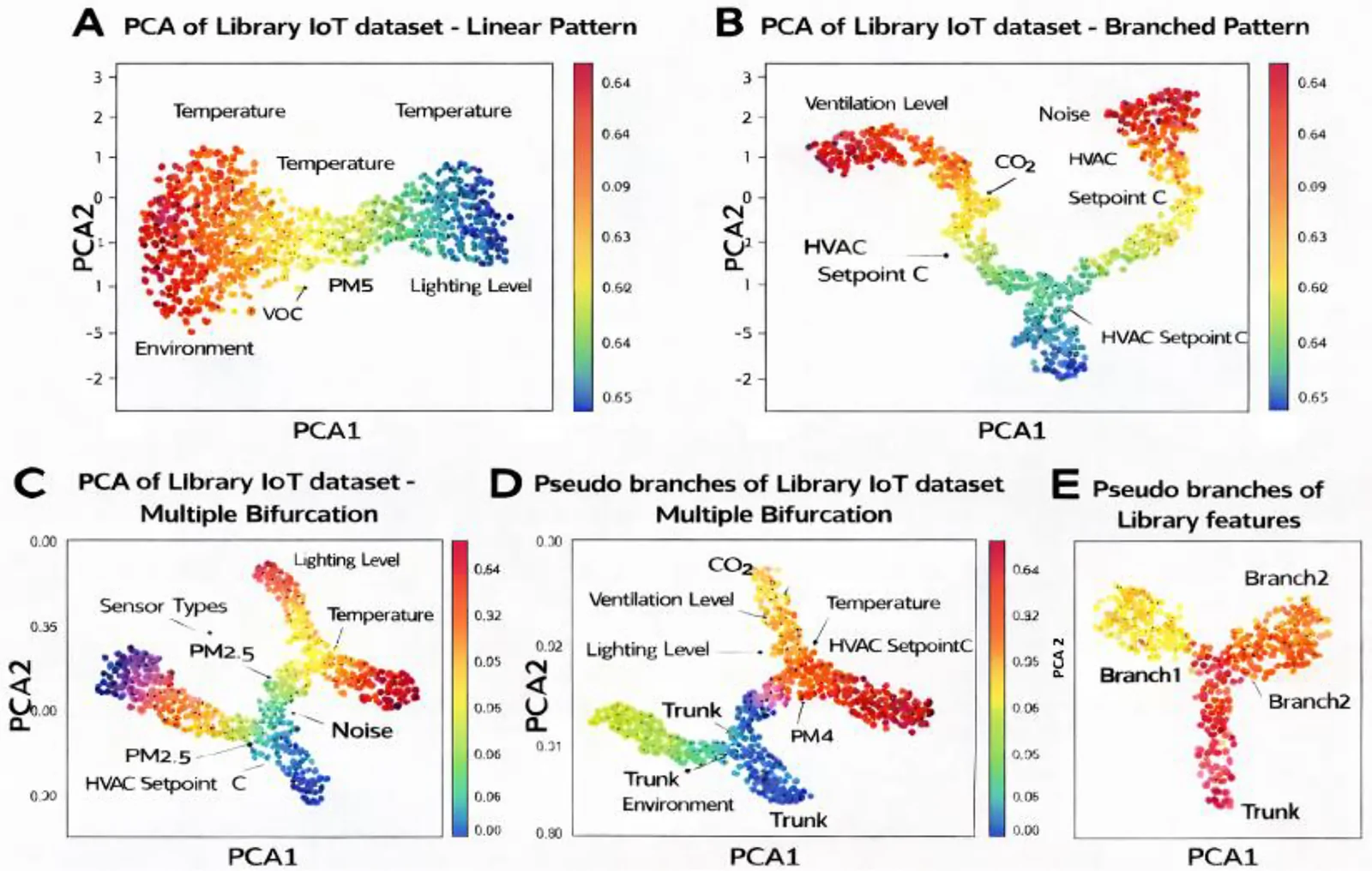
PCA-enabled feature discrimination highlighting adaptive smart library environmental state progression.

The smart library monitoring framework continuously collects environmental information through distributed sensors. Fig 5 illustrates the capability of PCA-based transformation to reveal hidden IoT feature relationships and dynamic environmental clusters for effective smart library management and adaptive control.

The CPA-RAdaBoost is an innovative model that utilizes the RF, AdaBoost, and CPA algorithms, which complement each other in their abilities to recognize environmental states. In order to use the most relevant features obtained from PCA, RF creates nonlinear relations between multiple sensors’ data using an ensemble of decision trees. Next, AdaBoost helps classify by emphasizing those environmental states that were misclassified before and borderline environmental states. Finally, CPA tunes the weights of the features and the values of hyperparameters such as the number of trees, the depth of trees, and the learning rate by optimizing the validation accuracy.

### RF-based environmental pattern recognition

RF-based methods used in indoor library environments perform heterogeneous sensor data processing using a group of decision trees (DTs) in order to detect complicated nonlinear relationships between factors like temperature, air quality indicators, lighting, acoustics, and occupancy characteristics. Such ability allows for reliable detection of environmental conditions and enables intelligent decisions concerning dynamic control [31]. The bagging technique is based on the use of many base learners in order to provide more accurate and stable predictions. Such an approach increases the generalization abilities of the supervised learning algorithm by averaging the results obtained from different training samples.

The procedure begins by constructing multiple training subsets from the original dataset containing **N observations** through random resampling techniques known as bootstrap sampling. Each generated subset maintains an equivalent size to the initial dataset; however, individual samples may appear repeatedly, while some original observations may not be selected during a particular sampling process. As represented in Equation (7), the bootstrap dataset is formulated as:


$$\begin{array}{c} C_{s}=\{(w_{\text{1}},z_{\text{1}}),(w_{\text{2}},z_{\text{2}}),...,(w_{m},z_{m})\} \end{array}$$ (7)

where $C_{s}$ represents the $s^{th}$ bootstrap-generated dataset used for training each decision tree (DT), and $\left(w_{\text{1}},z_{\text{1}}\right)$, $\left(w_{\text{2}},z_{\text{2}}\right)$, etc., denote the randomly selected input-output pairs obtained through sampling with replacement.

After training the individual base learners on these generated subsets, they are then used for predicting the values of the output of unseen data points. In cases of classification problems, the final prediction is made on the basis of majority voting, whereby the label having the maximum votes is taken as the predicted class label. In the case of prediction problems based on regression, the values of all the individual trees are combined to give the final predicted value. In the suggested library environment monitoring system, this prediction reflects the different states of the environment, such as “comfortable,” “moderately uncomfortable,” or “critical” on the basis of parameters like temperature, indoor air quality, illumination, and acoustics. The combination of predictions is given in Equation (8).


$$\begin{array}{c} \hat{z}=\frac{\text{1}}{C}\sum_{c=\text{1}}^{C}{\hat{z}}_{m}(W) \end{array}$$ (8)

${\hat{z}}_{m}(W)$ is used to denote the prediction produced by the $m^{th}$ decision tree, while C stands for the number of bootstrap samples used in ensemble learning.

RF is a very useful ensemble learning approach that can be used in classification as well as regression problems. Instead of using only one decision tree model for the prediction process, RF utilizes the predictions made by many DT models in order to increase robustness and to reduce prediction uncertainty. The function $e_{s}(w$ is a measure of the performance of each tree, where the interactions among different environmental factors can be easily understood. For example, the interaction between the occupancy and CO_2_ levels in determining indoor air quality or the impact of lighting and background noise levels on occupant comfort.

Prediction by each individual decision tree is given by Equation (9):


$$\begin{array}{c} e_{s}(w)=\frac{\text{1}}{|k_{s}(w)|}\sum_{j\in k_{e}(w)}z_{j} \end{array}$$ (9)

Equation (10) shows the total forecast produced by the RF model as follows:


$$\begin{array}{c} \hat{z}(w)=\sum_{s=\text{1}}^{S}e_{s}(w) \end{array}$$ (10)

Here S is the total number of decision trees present in the forest, whereas $k_{s}(w$ and $k_{e}(w$ are the selected feature subsets and prediction regions associated with each tree, respectively. After RF model recognizes the environmental state, the intelligent control actions will be provided for heating ventilation air conditioning (HVAC) system control, lighting control, and ventilation control. By repeatedly implementing this process, RF can realize the adaptation of indoor environmental conditions in order to provide comfort to users while reducing energy usage, thus creating a proper learning environment in different occupancy scenarios and changing outdoor environments. Fig 6 shows the ensemble learning scheme for intelligent environmental state prediction and control.

**Fig 6:**
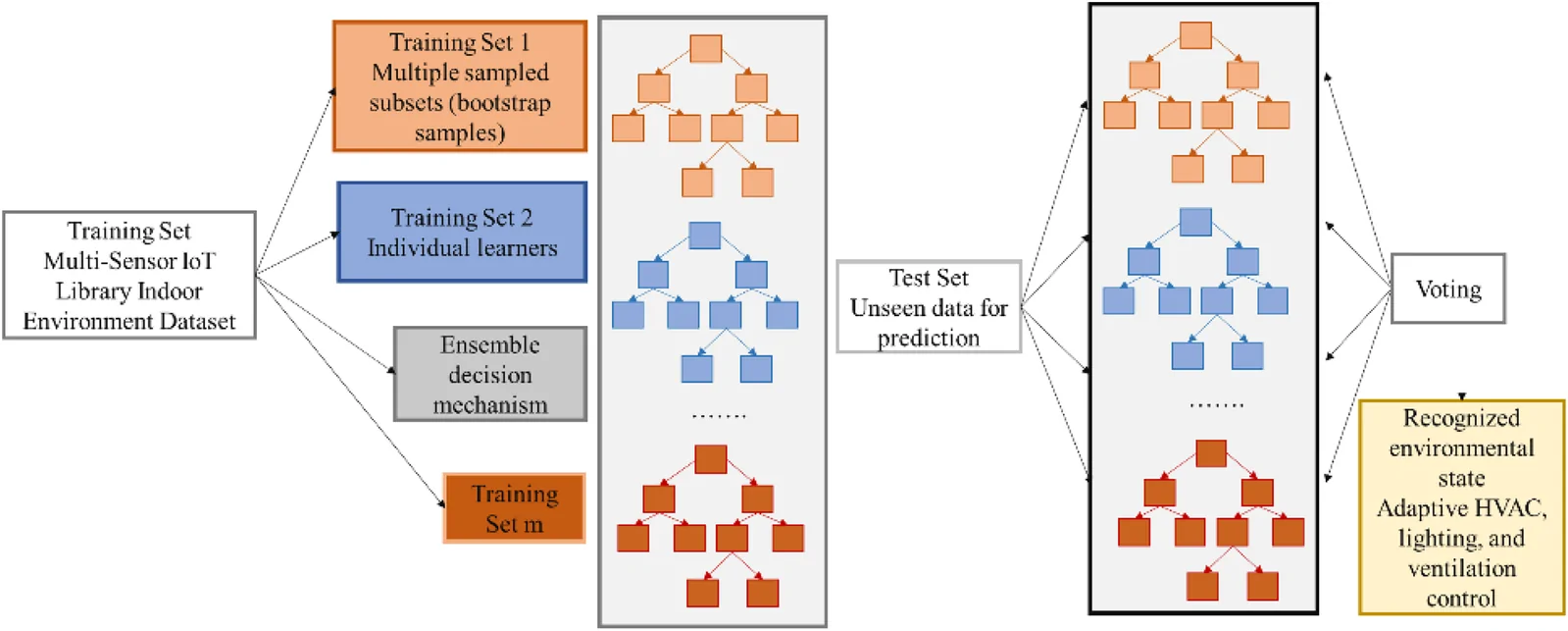
Ensemble methodology assisted by RF for predicting environment status in smart libraries.

### AdaBoost-based environmental condition classification

In intelligent libraries indoor environment conditions, AdaBoost works by combining several weak learners that are incrementally refined based on sensor measurements of the environment. It enhances the indoor conditions classification and helps in controlling HVAC systems, lighting, air flow, and noise control [32]. Being one of the efficient ensemble techniques, AdaBoost involves combining several base classifiers for building a strong prediction model based on environmental features $x_{\text{1}},x_{\text{2}},...,x_{m}$. It does so by modifying the weight of each weak learner to enhance the prediction accuracy while minimizing the chances of over-fitting the data. The overall structure of AdaBoost is shown in Fig 7, and the algorithm is as follows:

**Fig 7:**
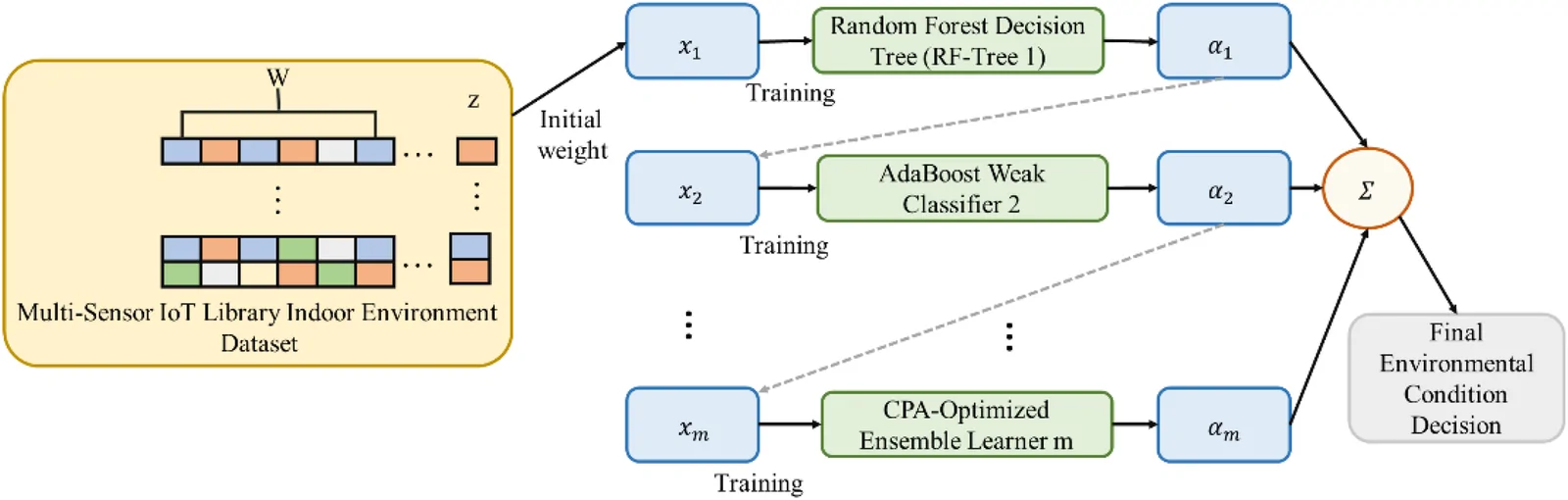
AdaBoost-guided ensemble approach for smart library environment forecasting and management.

At first, the error prediction is made based on the weak learner regression at stage s through $E_{s}$. The maximum error between the real and predicted environmental states can be computed using Equation (11).


$$\begin{array}{c} E_{s}=\text{max}|z_{j}-g_{s}(w_{j})|(j=\text{1},\text{2},...,n) \end{array}$$ (11)

Where n refers to the total number of observations, while w_{j} and z_{j} represent the sensor input properties and the environmental response values, respectively. The max function determines the maximum prediction error since variation in the input sensor reading may cause uncertainty or intermediary comfort readings.

Each sample’s deviation can be determined using the loss evaluation function $F_{s}$ which considers $\epsilon_{sj}$ as the sample error as shown in Equation (12).


$$\begin{array}{c} \epsilon_{sj}=\frac{|z_{j}-g_{s}(w_{j})|}{F_{s}} \end{array}$$ (12)

Overall coefficient of error for regression will then be calculated using Equation (13). In which, $\sum_{j=\text{1}}^{m}\;$ will represent the total contribution of all the selected samples, $X_{sj}$ will represent the relative weight of each sample while $\epsilon_{sj}$ will represent the error value of each sample.


$$\begin{array}{c} \epsilon_{s}=\sum_{j=\text{1}}^{m}X_{sj}\epsilon_{sj} \end{array}$$ (13)

Based on the calculated error coefficient, the proportion factor $\alpha_{s}$ for sample adjustment is determined according to Equation (14). The term $\text{1}-\epsilon_{s}$ reflects the influence of local environmental variations caused by measurement uncertainty and sensor disturbances.


$$\begin{array}{c} \alpha_{s}=\frac{\epsilon_{s}}{\text{1}-\epsilon_{s}} \end{array}$$ (14)

The distribution of training samples is modified iteratively through $X_{s+\text{1}}(w_{j})$, where the updated probability representation for subsequent boosting rounds is calculated using Equations (15) and (16). $Y_{s}$ denotes the normalization parameter, while $X_{s}(w_{j})$ represents the sample probability distribution at the current iteration.


$$\begin{array}{c} X_{s+\text{1}}(w_{j})=\frac{X_{s}(w_{j})}{Y_{s}}\alpha_{s}^{\text{1}-\epsilon_{sj}} \end{array}$$ (15)

$$\begin{array}{c} Y_{s}=\sum_{j=\text{1}}^{n}X(w_{sj})\alpha_{s}^{\text{1}-\epsilon_{sj}} \end{array}$$ (16)

The summation term $\sum_{j=\text{1}}^{n}\;$ provides the normalization operation, whereas $X(w_{sj})$ maintains stable weight distribution and preserves the probabilistic relationship among sensor observations during model optimization.

Finally, the combined AdaBoost predictor G(w) generates the final indoor environmental state estimation as represented in Equation (17).


$$\begin{array}{c} G(w)=\sum_{s=\text{1}}^{S}In(\text{1}/\alpha_{s})e(w) \end{array}$$ (17)

where e(w) represents the weighted output generated by the ensemble learners, and $\sum_{s=\text{1}}^{S}\;$ integrates the contribution of all boosting iterations. The term $In(\text{1}/\alpha_{s})$ adjusts the influence of each weak learner according to its prediction capability, enabling the library environment management system to maintain stable thermal comfort under changing occupancy levels and operational requirements.

### CPA-based feature and parameter optimization

The process of adaptive optimization of feature importance and parameter tuning of the algorithm is performed through mimicking the cooperative hunting strategies; therefore, the classification capabilities of the proposed system are improved. This allows the intelligent control system to take correct decisions in relation to various occupancy, comfort requirements, and indoor conditions [33]. The main concept of the colony predation algorithm is based on the predators’ cooperative hunting behavior where the information exchange helps them to capture their prey.

Equation (18) represents the updating mechanism of $W_{i}^{j}(s+\text{1})$, where the position of each search agent is modified by considering neighboring individuals to improve the identification of complex and transitional environmental conditions.


$$\begin{array}{c} W_{i}^{j}(s+\text{1})=W_{i}^{j}(s)+(\text{1}-q).((W_{\text{1}}(s)+W_{\text{2}}(s))/\text{2}) \end{array}$$ (18)

In this case, j is the dimensional index, i is the search agent number, and W_{1}(s) represents the position of the search agent at the moment it searches for the prey’s position in the j-th dimension. $W_{\text{2}}(s)$ is the position of the search agent derived from the next search iteration, while $W_{i}^{j}(s)$ is the position value of the search agent. The parameter q is the random coefficient between 0 and 1, and $W_{\text{1}}\;and\;W_{\text{2}}$ refer to the positions of the neighboring search agents that are close to the optimal point.

Equation (19) describes the prey movement strategy, where $W_{best}$ represents the optimal solution obtained during the search process. The generated solution W(s+1)distributes candidate solutions across different regions of the search space, allowing the optimization process to avoid premature convergence. The upper boundary ub and lower boundary lb maintain feasible parameter ranges, ensuring that the library environment achieves stable comfort regulation, reduced energy consumption, and reliable operation despite variations in user density and external disturbances.


$$\begin{array}{c} W(s+\text{1})=W_{best}-T.(ub-lb)+lb \end{array}$$ (19)

In Equation 19, $T_{\text{0}}$represents the energy level of prey, which controls the exploration intensity during optimization. The parameter b/M represents the left and right boundary distances, while s is a random coefficient between 0 and 1. $q_{\text{2}}$ indicates the prey position factor, and $T_{\text{0}}$ describes the current state of the searching agent. The cooperative searching behavior is further formulated in Equations (20) and (21), enabling the optimization model to capture minor environmental fluctuations such as gradual CO_2_ concentration variation, temporary acoustic disturbances, or short-term occupancy changes.


$$\begin{array}{c} T_{\text{0}}=b-s.(b/M) \end{array}$$ (20)

$$\begin{array}{c} T=\text{2}.T_{\text{0}}.q_{\text{2}} \end{array}$$ (21)

Equation (22) defines the infiltration behavior of predators after the prey distribution phase. The term $f^{k}.tan(\pi /\text{4}k)$ controls the movement intensity of search agents, enabling the proposed system to maintain a suitable balance between indoor comfort, energy utilization, and adaptive response. The factor 2T.C supports dynamic adjustment of environmental control actions according to occupancy variations and unexpected environmental changes while preserving system stability.


$$\begin{array}{c} W(s+\text{1})=W_{best}-\text{2}T.C.f^{k}.tan(\pi /\text{4}k) \end{array}$$ (22)

$$\begin{array}{c} C_{\text{1}}=|\text{2}q_{\text{4}}.W_{rand}-W(s)| \end{array}$$ (23)

In Equation (23), $C_{\text{1}}$ defines the movement distance between the current search agent and the randomly selected prey position. $W_{rand}$ represents the randomly selected location generated from the prey population, whereas $\text{2}q_{\text{4}}$ controls the influence of the neighboring prey position during the search operation. The final position updating rule is expressed in Equation (24), where two alternative searching behaviors are selected based on the value of $|q_{\text{6}}|$.


$$\begin{array}{c} W(s+\text{1})=\left\{ \begin{array}{cc} O_{nearest}, & |q_{\text{6}}|\leq \text{1}\\ W_{rand}-T.C_{\text{1}}, & |q_{\text{6}}|>\text{1} \end{array}\right. \end{array}$$ (24)

Equation (24) demonstrates the adaptive switching capability of CPA between exploitation and exploration phases. When $|q_{\text{6}}|\leq \text{1}$, the nearest optimal solution is selected to maintain stable environmental regulation and energy-efficient operation within the library environment. Conversely, when $|q_{\text{6}}|>\text{1}$, the random exploration mechanism is activated to rapidly respond to dynamic indoor changes. Therefore, CPA provides an intelligent optimization strategy capable of achieving robust, responsive, and adaptive environmental monitoring and control specifically designed for smart library spaces. Fig 8 demonstrates the CPA algorithm adaptation for library environment regulation. The proposed approach procedure is presented in Algorithm 1.

**Fig 8:**
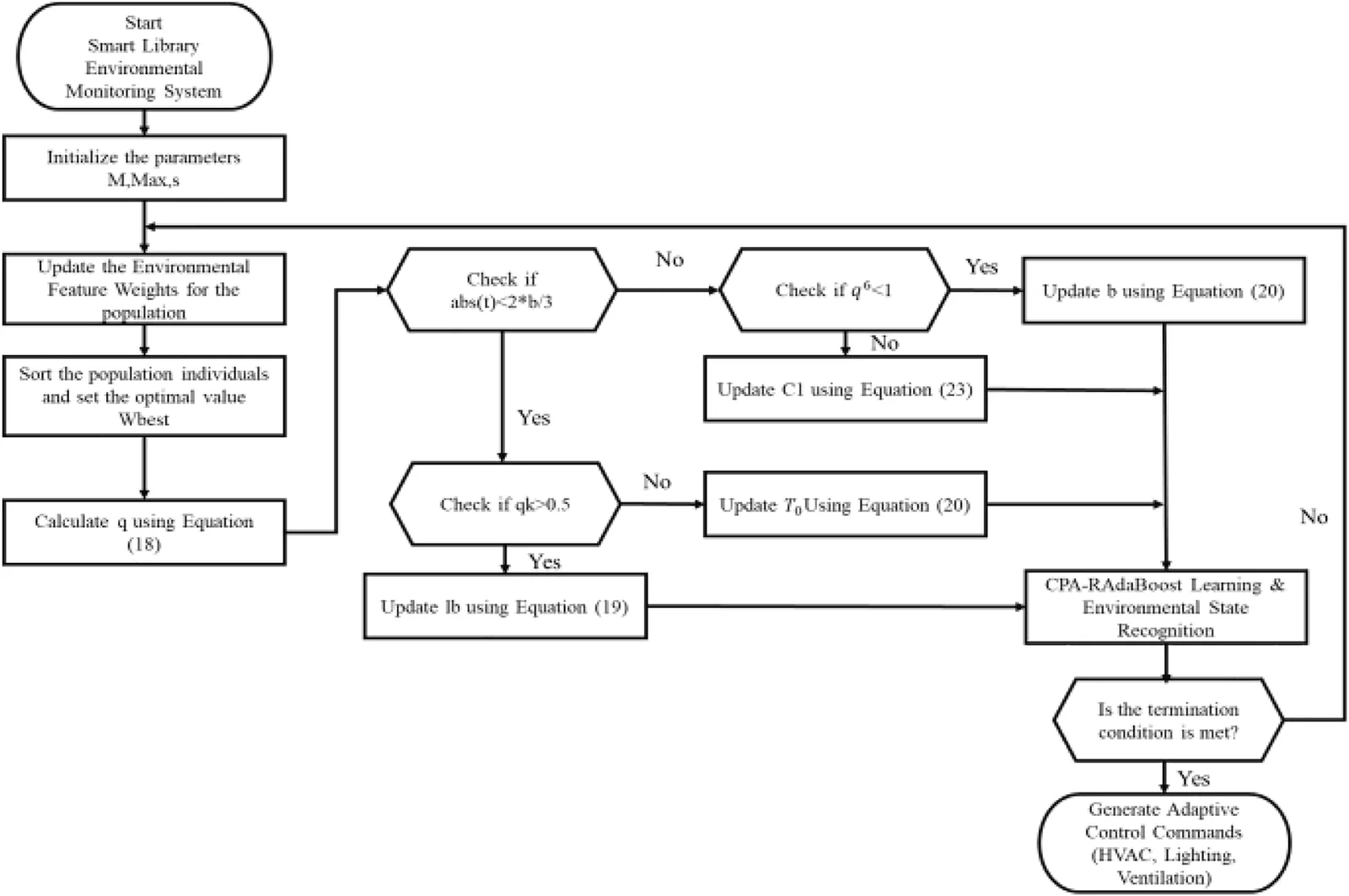
CPA-guided framework for dynamic intelligent library climate condition detection and regulation.

### AI-based library environment monitoring and control

The designed scheme is meant to introduce an intelligent feedback control system for the indoor library environment. IoT-enabled sensing nodes that have several types of sensors gather real-time data about the temperature, humidity, CO2 concentration, illuminance, and occupancy of different parts of the library. Principal Component Analysis (PCA) will be employed to identify the most important features from the dataset and remove redundant features while keeping important variations in the dataset. Artificial Intelligence-based CPA-RAdaBoost classification method will be created to determine the current environmental condition, in which CPA finds the optimal combination of features and model parameters for better performance.

**Operational Control Approach:** The states generated by the CPA-RAdaBoost algorithm in the environmental domain are then translated into rule-based control actions to operate the HVAC, ventilation, and lighting systems accordingly. The Comfortable state does not change the operating conditions, while the Slightly Uncomfortable and Critical states result in increasingly more aggressive control actions. Following the actuation step, new readings from the IoT sensors are recorded and used in state prediction, resulting in a feedback loop between sensing, prediction, and control. In our approach, we use a rule-based control approach without using any physical/dynamic building model.

To enhance methodological transparency and reproducibility, the designed framework operates through a sequence of steps. PCA selects the most informative features that are employed to develop a Random Forest classifier. AdaBoost enhances the classification by highlighting the misclassified examples, whereas CPA maximizes the weights of the features and important hyperparameters prior to training the final model. The enhanced CPA-RAdaBoost model is finally applied to environmental state detection and adaptive control. Fig 9 illustrates the developed CPA-RAdaBoost model for intelligent and dynamic library environment regulation.

**Fig 9:**
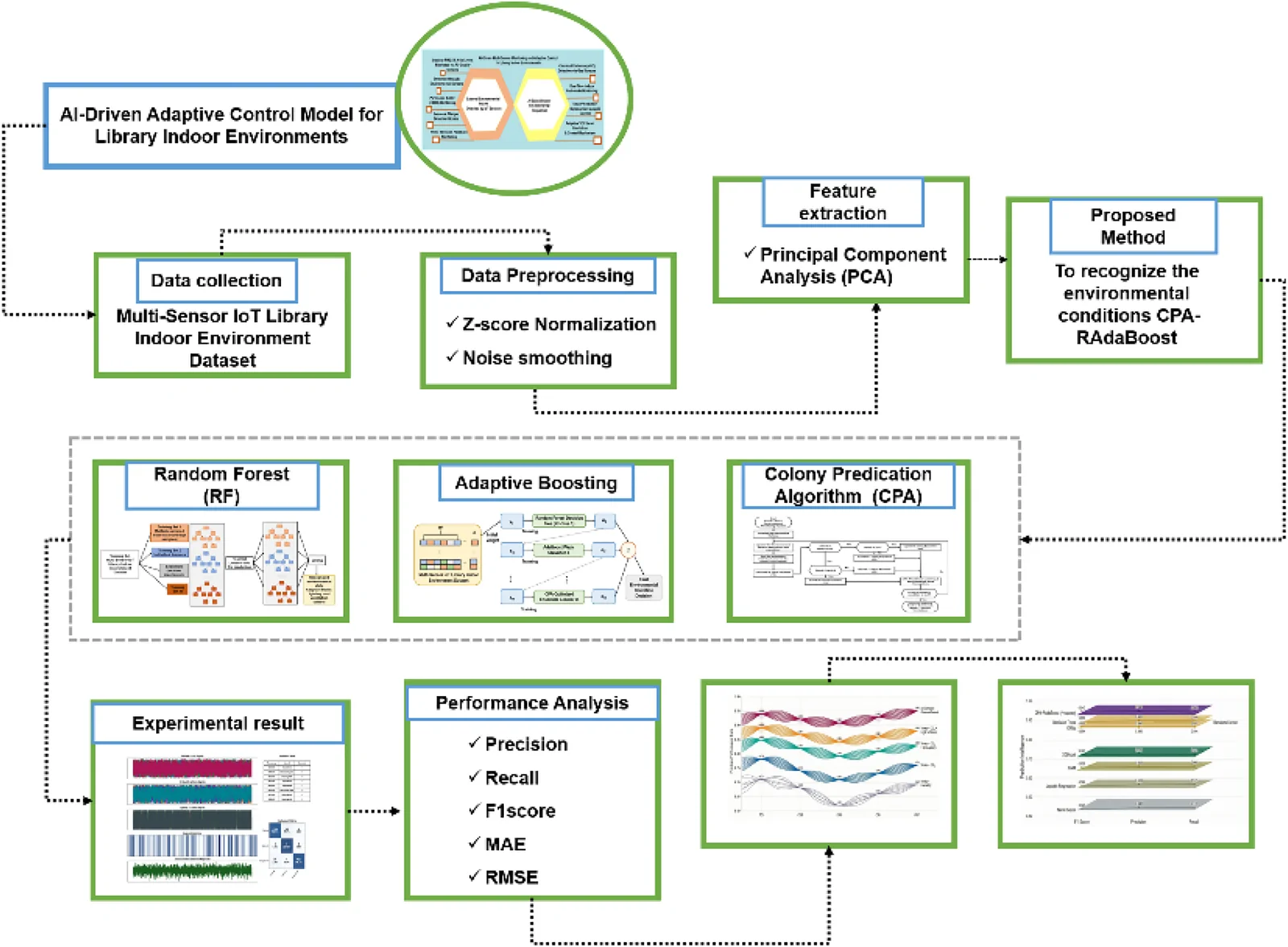
Comprehensive pipeline for the developed CPA-RAdaBoost-driven library climate regulation and management framework.

This methodology operates on a sequential manner within one united system. This procedure begins from the preprocessing phase, where feature extraction takes place using PCA. Next, CPA identifies the best possible weights for the features and parameters for Random Forest. Further, Random Forest builds nonlinear relationships among environmental features, while AdaBoost determines incorrectly classified objects. Finally, the outcomes obtained are converted into adaptive control commands for HVAC, lighting, and ventilation systems.

The proposed framework can also be used in the practice of smart library buildings. The IoT nodes will continuously measure environmental data indoors and transmit it to the edge computing nodes, which will preprocess the data, and artificial intelligence will make decisions based on that. Due to the modular nature of the framework, any new sensors or areas/floors of the library building can be incorporated into the system without changes in the CPA-RAdaBoost framework, thereby making it scalable. This framework is capable of being integrated with Building Management System (BMS) and adjusting HVAC, lighting, and ventilation systems.

**Algorithm 1: CPA-RAdaBoost**

Input:

  Sensor dataset D = {Temperature, CO2, AirQuality, Light, Noise, Occupancy}

      Number of trees T

  Number of boosting rounds S

    Population size P

     Max CPA iterations I

  Feature bounds [ub−lb]

Output:

      Predicted environmental state Z

  Adaptive control commands for HVAC, lighting, and ventilation

Step 1: Data Preparation

Normalize sensor readings

Label environmental states as:

     0 → Comfortable

    1 → Slightly Uncomfortable

  2 → Critical

Split the dataset into training and testing sets

Step 2: Random Forest Initialization

For t = 1 to T do:

  $Create\;a\;bootstrap\;sample,\;C_{s},\;from\;training\;data$

  $\;Train\;DT\;Tree\;\hat{z}\;using\;random\;feature\;subsets$

End For

Step 3: AdaBoost−Based Tree Weighting

$Initialize\;sample\;weights\;E_{s}\;for\;all\;samples$

For s = 1 to S do:

    Train a weak learner using weighted samples

  Predict the environmental state for all samples

  $Compute\;prediction\;error\;g_{s}$

  $\;Compute\;learner\;weight\;g_{s}$

  $\;Update\;sample\;weights\;\left(w_{j}\right)$

  $\;Normalize\;X_{sj}$

End For

Step 4: CPA Initialization

$Initialize\;population\;W_{i}^{j}(s+\text{1}randomly\;within\;bounds$

$Evaluate\;the\;fitness\;of\;each\;W_{\text{1}}(susing\;RF-AdaBoost\;accuracy$

$Identify\;the\;best\;solution\;W_{\text{2}}(s)$

Step 5: Final Model Construction

$Apply\;optimized\;feature\;weights\;and\;hyperparameters\;from\;T_{\text{0}}$

Train the final Random Forest with AdaBoost weighting

Step 6: Environmental State Prediction

For each tree t do:

  $\;Predict\;environmental\;state\;Y_{s}$

End For

Aggregate predictions using majority voting/averaging

Obtain the final predicted state Z

Step 7: Adaptive Control Decision

If Z == Comfortable then

  Maintain current HVAC, lighting, and ventilation settings

Else if Z == Slightly Uncomfortable then

  Adjust airflow, lighting intensity, and temperature slightly

Else

  Trigger aggressive control actions for safety and comfort

End If

Return Z and control commands

### Ethical approval statement

This study did not involve human experiments and used a publicly available, anonymized dataset containing no personally identifiable information.

## Results and discussion

The experimental test is carried out in the specified library area fitted with IoT-based sensors to constantly sense variations. The sensed information is then forwarded to the edge computing system, where preprocessing of the data is conducted by scaling and interference elimination via Python 3.x computational packages such as Pandas, NumPy, SciPy, Scikit-learn (PCA, Random Forest, and AdaBoost), as well as through the designed CPA technique. The CPA-RAdaBoost classifier evaluates the state of the indoor environment, detects the present comfort state, and creates appropriate control actions for HVAC, illumination, and ventilation equipment. The performance of the proposed solution is measured via F1-score and response time. Table 1 shows the parameter settings that have been optimized for efficient and energy-aware indoor environment management.

**Table 1:** Optimized parameter configuration of CPA-driven AdaBoost RF for reliable indoor library condition classification.

Components	Hyperparameter	Symbol	Value / Range	Final Selected Value
RF	Number of trees	T	100−300	200
	Feature subset size	${\hat{z}}_{m}$	−	√M
	Minimum samples per leaf	$w_{\text{2}}$	1-5	2
	Maximum tree depth	$z_{\text{2}}$	10−30	20
AdaBoost	Learning rate	η	0.01−0.1	0.05
	Initial sample weight	$z_{j}$	–	1/N
	Number of boosting rounds	S	30−100	50
CPA	Population size	P	20−50	30
	Maximum iterations	I	50−100	60
	Exploration probability	q	0.3−0.7	0.5
	Lower bound	lb	−	0
	Upper bound	ub	−	1
Control System	Environmental classes	Z	−	{0,1,2}

42 is the random seed; 30 candidate solutions are randomly initiated within the given bounds for the CPA population; a maximum of 60 iterations is the stopping requirement.

The dataset employed in this study is the Multi-Sensor IoT Library Indoor Environment Dataset. This is a dataset containing 2,745 records that were recorded through different zones of an academic library in China. This dataset is composed of 18 features including occupation, environment, sensors, control, and context attributes. The environmental parameters will consist of temperature, relative humidity, carbon dioxide concentration, illuminance, noise, PM2.5 concentration, and VOCs. Number of occupants and sensor type are sensing features, while HVAC set point, ventilation, and light levels define the environment control parameters. Contextual features include time stamp, zone ID, building ID, and floor ID. The target feature is the state of the environment, known as Overall_Environment_State. Training and test data is divided such that there is 80% training and 20% testing data. The dataset is publicly available through Kaggle. https://www.kaggle.com/datasets/colabsss/multi-sensor-library-indoor-environment-dataset.

Data were acquired from four functional indoor zones: Reading Hall, Digital Laboratory, Stack Area, and Discussion Zone. The monitoring period spans from 1 January 2024–10 January 2024 with continuous time-stamped measurements. The dataset contains 2,745 observations and includes environmental variables (temperature, humidity, CO_2_, PM2.5, VOC, illuminance, and noise), occupancy information, HVAC setpoints, ventilation levels, lighting settings, and sensor metadata. These heterogeneous measurements provide comprehensive information for intelligent indoor environmental assessment and control.

### Case study description

Include:

- Country: China
- Facility: Academic Library
- Monitoring period: 1–10 January 2024
- Number of records: 2,745
- Four monitored zones
- Multi-floor monitoring
- Multiple IoT sensors
- Indoor environmental monitoring
- HVAC-assisted environmental control

According to the confusion matrix, the developed CPA-RAdaBoost algorithm is capable of classifying indoor environments as being comfortable, slightly uncomfortable, or critical with minimal error rate, thus proving its efficiency in assisting intelligent environmental monitoring and automatic HVAC control of the smart library system, as shown in Fig 10.

**Fig 10:**
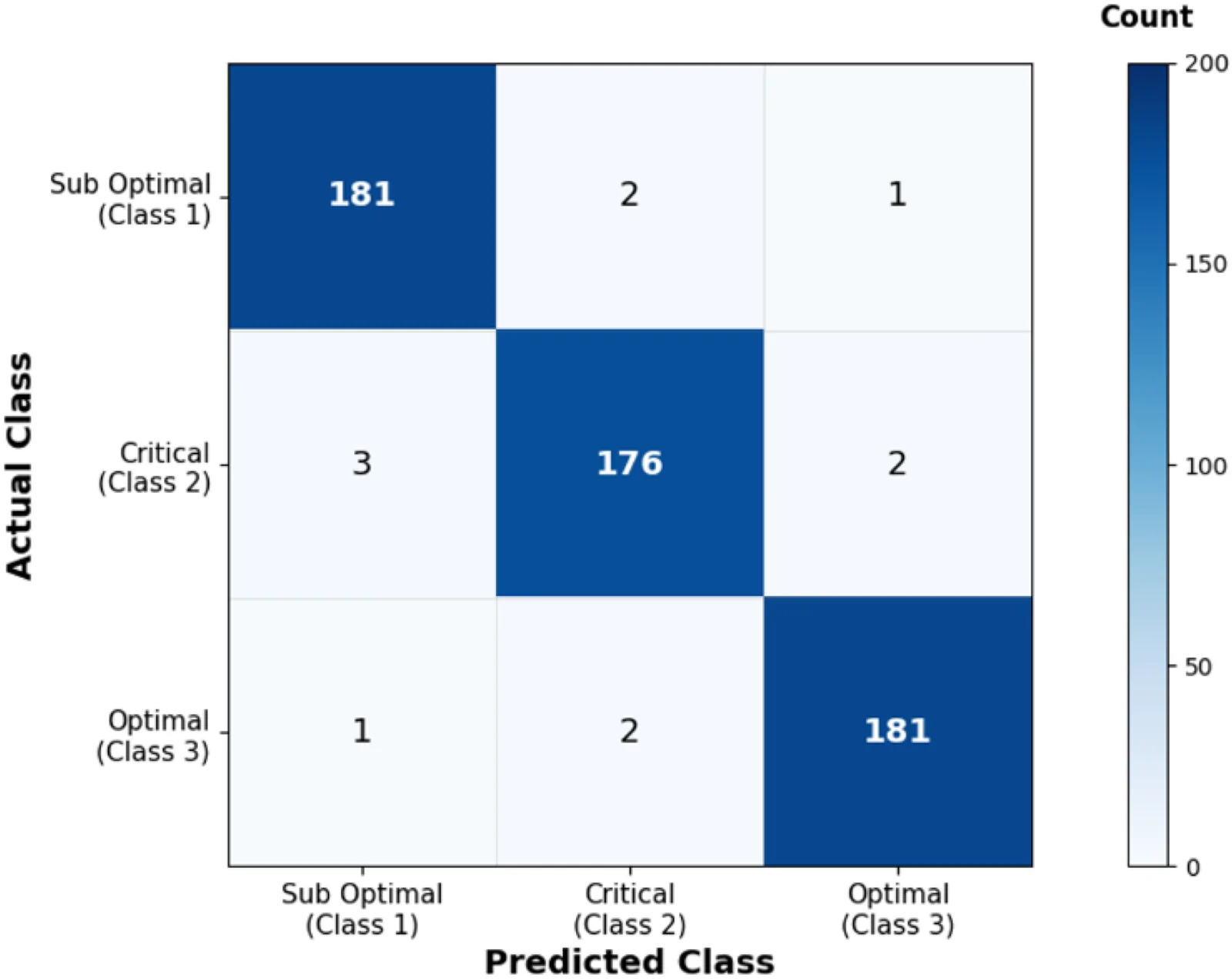
Confusion matrix of the proposed CPA-RAdaBoost model for multiclass indoor environmental state classification.

### Edge-level sensor aggregation for reduced latency

The raw data obtained from sensors are transferred to edge computing devices that lie close to the sensors and are placed within the monitoring framework. Edge processing helps in the fast analysis of data by facilitating the coordination of data from heterogeneous sensor sources and decreasing the delay due to reliance on cloud servers. Fig 11 shows the edge-cloud processing procedure used for the adaptive control of smart library environments.

**Fig 11:**
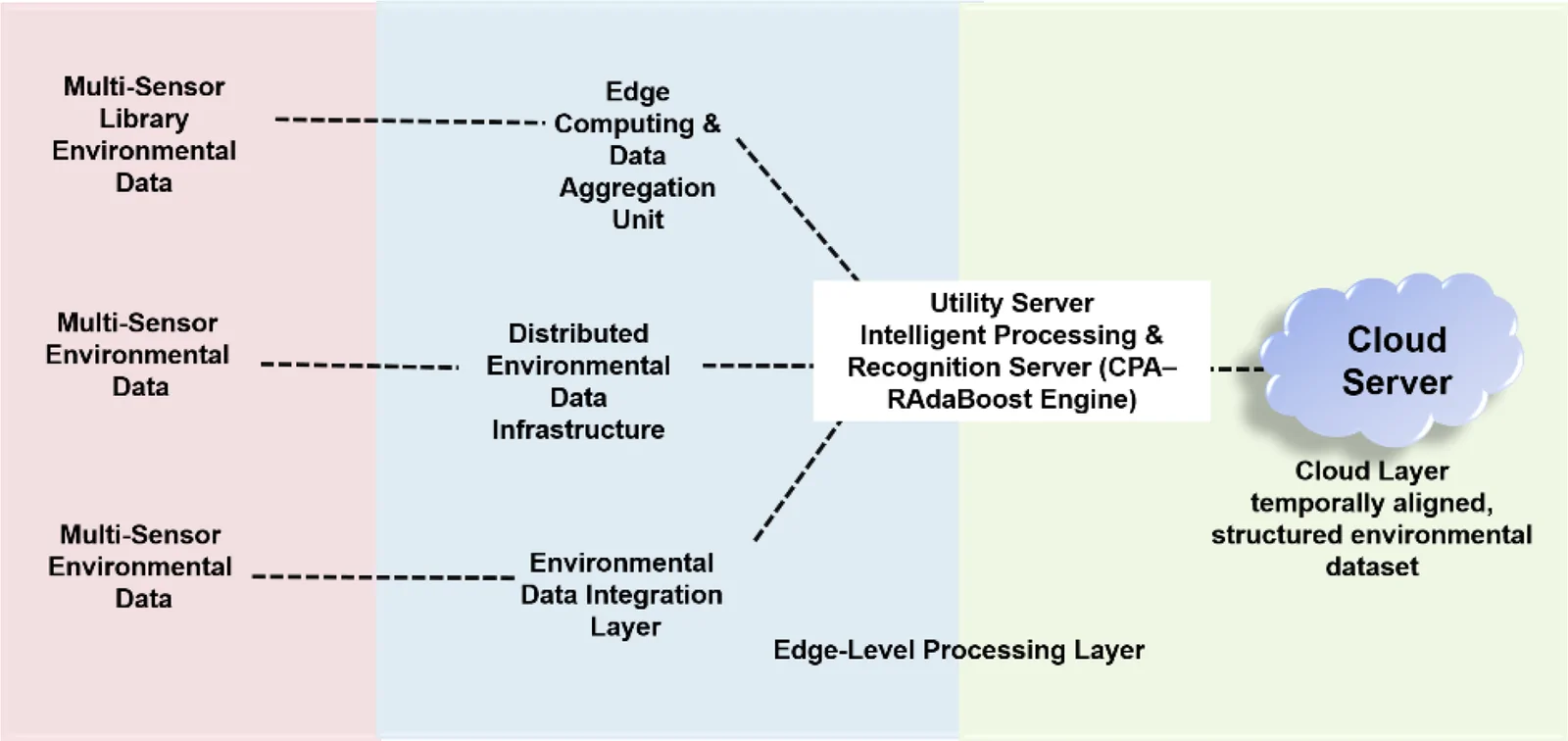
Cloud-integrated architecture for autonomous smart library environmental monitoring and control.

A visualization of the processed environment data for the adaptive control system of multi-sensor IoT-based library management is shown in Fig 12. Sensor parameters include temperature, CO_2_ content, noise pollution, PM2.5, VOCs, illumination levels, and digital lab, stack area, and discussion area. The combination of PCA with feature selection and the CPA-RAdaBoost classification algorithm provides an effective way to identify states of the indoor environment to enable intelligent library management using dynamic HVAC, airflow, and lighting control systems.

**Fig 12:**
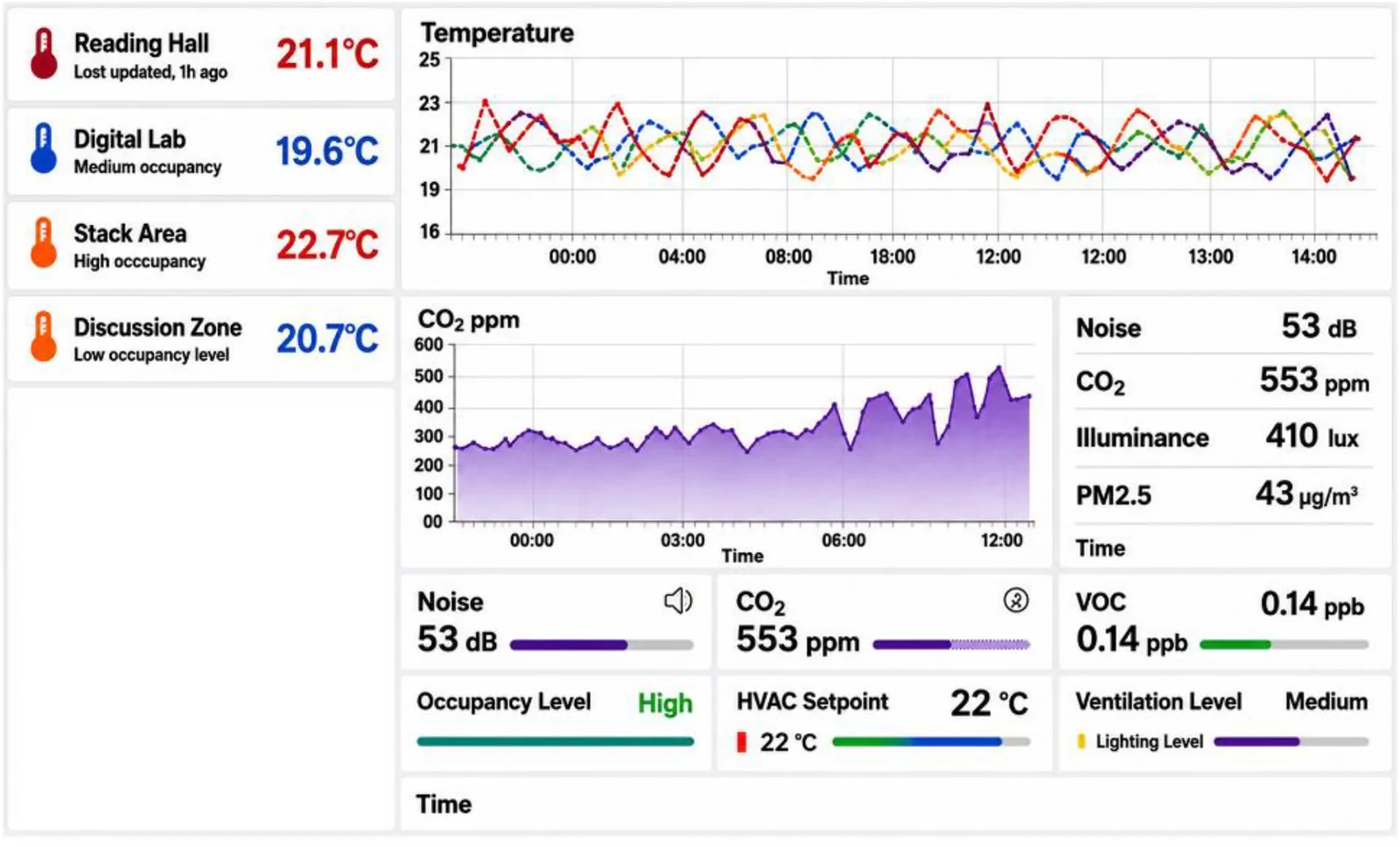
Unified IoT environmental data analysis facilitating adaptive intelligent library control optimization.

The accuracy and loss plots indicate a convergence pattern of the proposed CPA-RAdaBoost model. The training and testing accuracy is increasing in a steady manner, while the loss plots are decreasing steadily without much divergence, demonstrating effective learning, fast convergence, and good generalization capabilities of intelligent indoor environment monitoring and HVAC control, as illustrated in Fig 13.

**Fig 13:**
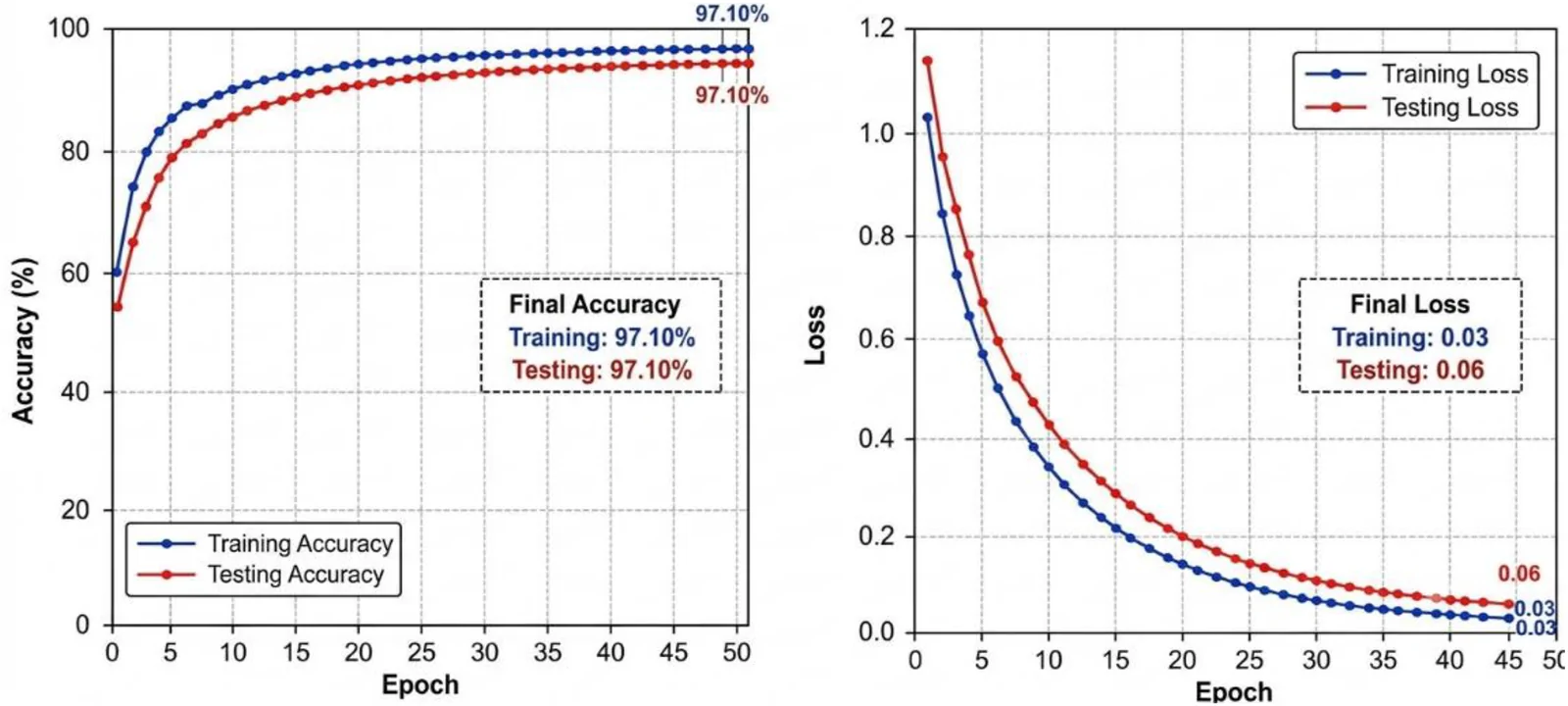
Training and validation accuracy and loss curves of the CPA-RAdaBoost model across boosting rounds.

Fan Comfort Index ↑ (FCI): This evaluates occupant comfort by analyzing the influence of temperature variation and airflow adjustment. CO2 Sensor Stability ↑ (CSS): Assesses the consistency and reliability of CO2 monitoring performance under changing indoor conditions. Energy Saving Score ↑ (ESS): Evaluates the capability of HVAC and lighting systems to optimize energy consumption during operation. Comfort Regulation Index ↑ (CRI): Represents the effectiveness of maintaining balanced and stable indoor environmental comfort. Fusion Response Fidelity ↑ (FRF): Indicates the accuracy of integrated multi-sensor data processing for responsive adaptive control, as presented in Table 2.

**Table 2:** Comparison study of performance between different sensor fusion methods for adaptive control using AI in libraries.

Sensor Fusion	FCI ↑	CSS ↑	ESS ↑	CRI ↑	FRF ↑
Temp + Humidity	0.71	0.68	0.62	0.66	0.69
*Temp + CO*_*2*_	0.78	0.74	0.71	0.73	0.76
*Temp + CO*_*2*_ *+ Occupancy*	0.85	0.82	0.79	0.81	0.83
*Temp + CO*_*2*_ *+ Light + Noise*	0.89	0.87	0.84	0.86	0.88
All Sensors (Proposed Fusion)	0.94	0.92	0.91	0.93	0.95

The AUC–ROC curves confirm the highly discriminative nature of the proposed CPA-RAdaBoost classifier in classifying the three types of indoor environment states, including Comfortable, Slightly Uncomfortable, and Critical. Fig 14 shows a strong macro-average AUC value of 0.992, confirming the highly accurate classification of the proposed model.

**Fig 14:**
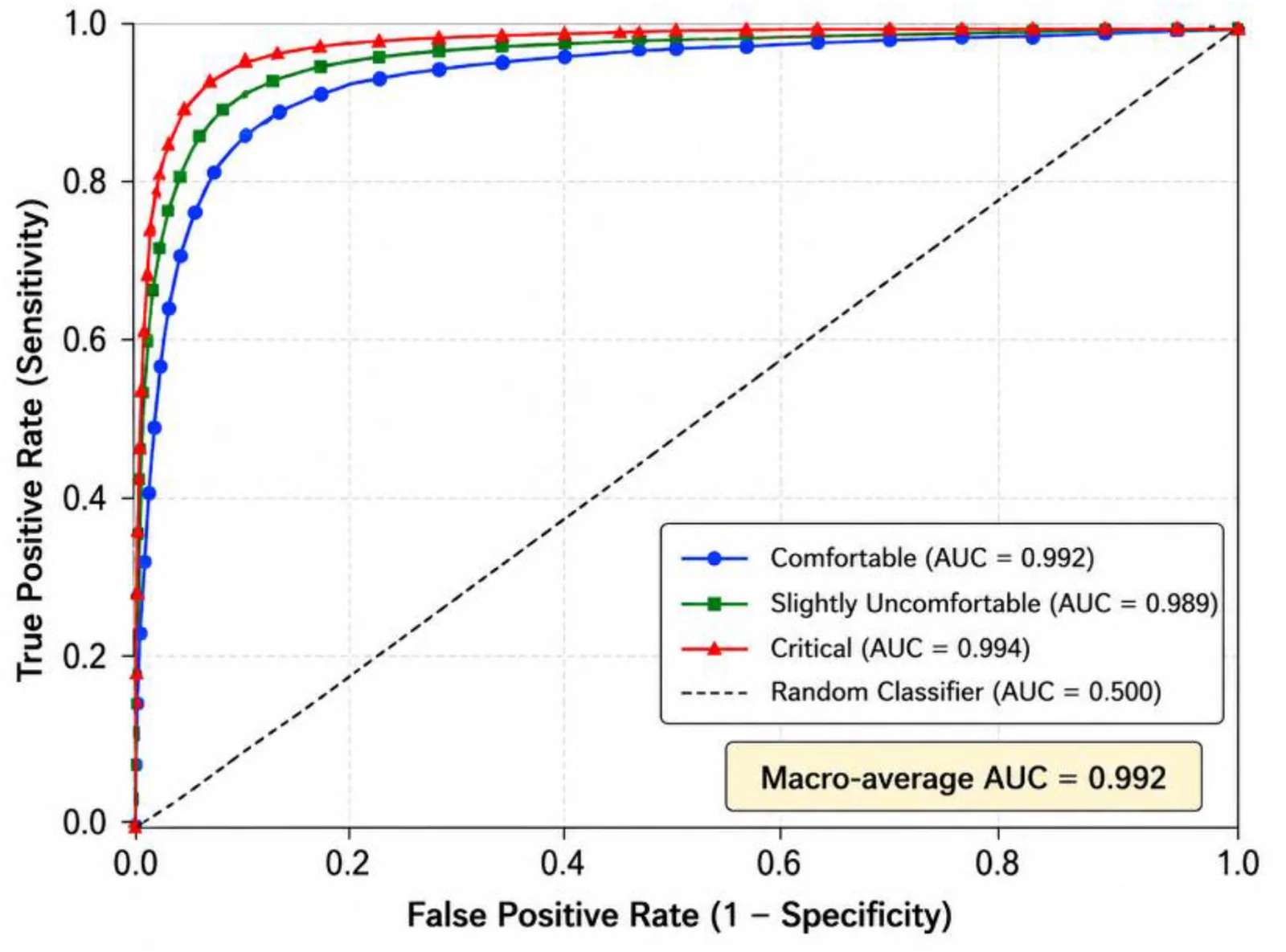
ROC curves of the CPA-RAdaBoost model for the three environmental states.

Maximum Data Rate of Sensors kbps: The highest possible rate of data transmission by IoT devices in terms of collecting environmental data. Packet Loss (%): Measures the level of stability in communication of wireless sensors; less packet loss will result in reliable automation controls. Average Latency (ms): Indicates the amount of time taken to collect data and initiate control actions in response to the collected data. Control Accuracy (%): The performance of HVAC, lighting, and ventilation systems in maintaining required environmental parameters. Performance assessment of the adaptive control system in multi-sensor IoT is shown in Table 3.

**Table 3:** Performance indicators of multi-sensor IoT adaptive control system.

Library Zone	Peak Sensor Data Rate (kbps)	Packet Loss (%)	Avg. Latency (ms)	Control Accuracy (%)
Reading Hall	45.8	1.1	52	96.9
Digital Lab	42.3	0.9	48	97.2
Stack Area	38.7	0.8	45	97.4
Discussion Zone	40.1	1.3	50	96.7
Overall Environment System	41.7	1.03	49	97.1

The performance metrics assess the control of smart library environmental conditions. Average Power Consumption (W) calculates the electrical energy consumed by the HVAC system and the lighting system, whereas the daily energy demand (kWh) measures the degree of energy optimization. The mean temperature fluctuation (°C) measures the stability of indoor temperature, and the mean adaptation delay (s) measures the capacity of the AI controller to quickly adapt to changes in occupancy and environmental factors. Table 4 shows the evaluation of AI-driven intelligent regulation for optimizing energy consumption, indoor comfort, and operational response in libraries.

**Table 4:** AI-enabled intelligent regulation optimizes library energy usage, indoor comfort, and operational response under varying occupancy conditions.

*Occupancy Level*	*Avg. Power* *Consumption* (*W*)	*24* *Hour Energy* (*kWh*)	*Avg. Thermal* *Rise* (*°C*)	*Avg. Response* *Time* (*s*)
Low (≤20% seats)	32.5	0.78	3.1	0.71
Medium (21−60% seats)	41.8	1.01	3.8	0.64
High (≥61% seats)	49.3	1.19	4.5	0.61
Overall	41.2	0.99	3.8	0.65

Table 4 shows that power consumption and thermal rise increase with occupancy (32.5 W to 49.3 W and 3.1 °C to 4.5 °C from low to high occupancy), while the response time decreases slightly (0.71 s to 0.61 s), confirming stable adaptive control under varying loads.

The Mean Squared Error (MSE) calculates the average squared difference between the observed and predicted sensor values, as expressed in Equation (25). It provides a quantitative measure of prediction error.


$$\begin{array}{c} MSE=\frac{\text{1}}{n}\sum_{i=\text{1}}^{n}(y_{i}-\hat{y_{i}}{)}^{\text{2}} \end{array}$$ (25)

Prediction error is quantified using MSE and RMSE (Equations 25–26), where $y_{i}$ and $\hat{y_{i}}$ are the observed and predicted sensor values, and n is the number of samples.

**Root Mean Squared Error (RMSE)** is obtained by taking the square root of MSE, converting the error value back into the original measurement scale, as represented in Equation (26).


$$\begin{array}{c} RMSE=\sqrt{\frac{\text{1}}{n}\sum_{i=\text{1}}^{n}(y_{i}-\hat{y_{i}}{)}^{\text{2}}} \end{array}$$ (26)

RMSE indicates the practical difference between predicted and actual environmental sensor observations, providing insights into the reliability of AI-driven adaptive environmental regulation and control performance.

Table 5 shows that the proposed model lowers both MAE and RMSE relative to MCIMS [29] with CPA-RAdaBoost for all four variables; for example, temperature MAE from 0.27 to 0.22 and humidity RMSE from 1.73 to 1.35.

**Table 5:** Evaluation of MCIMS versus our developed approach for precise indoor climate variable forecasting within libraries.

Parameters	MAE MCIMS [29]	MAE CPA-RAdaBoost- [Proposed]	MCIMS RMSE [29]	RMSE CPA-RAdaBoost- [Proposed]
$Temperature\;({\;}^{\circ }C)$	0.27	0.22	0.31	0.26
Humidity (%)	1.45	1.10	1.73	1.35
Light (lux)	5.8	4.9	6.4	5.2
*Dust* (*µg*/*m*^3^)	4.6	3.9	5.1	4.3

### Performance and generalization analysis

The Multi-Sensor IoT Library Indoor Environment Dataset (2,745 instances) was randomly shuffled and split into 80% for training (2,196 instances) and 20% for testing (549 instances) through stratified sampling. All preprocessing steps, such as Z-score normalization, Savitzky-Golay filter, and PCA, were trained on the training data before being applied to the test data to avoid any potential data leakage. CPA optimized the hyperparameters of the Random Forest and AdaBoost classifiers on the training data, whereas the testing set was held out for final evaluation of the models’ performance. To measure the robustness and generalization ability of the models, five-fold stratified cross-validation was done on the training data, and average values of Accuracy, Precision, Recall, and F1-score along with 95% confidence intervals are shown in Table 6 below.

**Table 6:** Five-fold cross-validation results of the proposed CPA-RAdaBoost model.

Fold	Accuracy (%)	Precision (%)	Recall (%)	F1-Score (%)
Fold 1	97.7	98.0	97.9	97.8
Fold 2	98.0	97.8	98.1	97.9
Fold 3	97.8	98.1	97.7	98.0
Fold 4	98.1	97.9	97.8	97.7
Fold 5	97.9	97.7	98.0	98.1
Mean	97.9 ± 0.16	97.9 ± 0.16	97.9 ± 0.16	97.9 ± 0.16

The five-fold cross-validation method is a technique used in statistical validation, where the data set is split into five equal parts. In each case, four parts are used to train, and one part is used for validation, and this process is done five times such that each part has been used once as the validation part. This technique helps assess the ability of the model to generalize.

The stability of the CPA-RAdaBoost model that was developed is evidenced by Table 6, where results were obtained using five-fold cross-validation. The model attained 97.9 ± 0.16% for the accuracy measure, 97.9 ± 0.16% for the precision metric, 97.9 ± 0.16% for the recall parameter, and 97.9 ± 0.16% for the F1 score.

### Ablation study

In an ablation study, the contribution of different components of the proposed model is evaluated by eliminating one by one all the components to observe their impact on the entire system. The impact of different modules can be observed in similar experimental settings, which proves the need for each component of the final model

Ablation study for the proposed model is illustrated in Table 7. Random Forest has good baseline performance, whereas AdaBoost has better performance in classifying environment states by focusing on the incorrectly classified environment states. Combining CPA with Random Forest results in even better optimization of the features and model convergence. The overall performance of CPA-RAdaBoost model is outstanding in F1-Score (0.979), Precision (0.979), and Recall (0.979) with minimal testing time (0.010 s).

**Table 7:** Ablation analysis demonstrating the contribution of each component in the proposed CPA-RAdaBoost model.

Model Architecture	F1-Score	Precision	Recall	Training Time (s)	Test Time (s)
Random Forest	0.899	0.901	0.899	30.45	0.12
AdaBoost	0.914	0.917	0.912	29.36	0.10
CPA + Random Forest	0.928	0.931	0.926	28.94	0.080
Random Forest + AdaBoost	0.936	0.939	0.934	28.63	0.060
CPA-RAdaBoost [Proposed]	0.979	0.979	0.979	17.12	0.010

### Comparative analysis with retrained models

The comparative analysis is conducted to evaluate the effectiveness of the proposed CPA-RAdaBoost model against conventional and advanced machine learning approaches, including MCIMS, KNN, SVM, Naive Bayes, Random Forest, XGBoost, CatBoost, Logistic Regression, LightGBM, Decision Trees, ELM, GPR, and DBN. All models were retrained and evaluated using the Multi-Sensor IoT Library Indoor Environment Dataset under identical data preprocessing, training-testing splits, hyperparameter optimization procedures, and experimental settings. This consistent evaluation protocol ensures a fair comparison of classification accuracy, computational efficiency, and real-time performance for temperature environment classification and ventilation control, thereby demonstrating the robustness and superiority of the proposed CPA-RAdaBoost framework.

Table 8 presents a comparison between the retrained accuracy of the traditional and the proposed ML methods used to classify the temperature environment category. LightGBM, CatBoost, and DBN perform well, but the proposed CPA-RAdaBoost performs better in terms of Precision (0.979), Recall (0.979), and F1 score (0.979).

**Table 8:** Machine learning models for temperature environment classification using the Multi-Sensor IoT Library Indoor Environment dataset.

Dataset	Method	Precision	Recall	F1-Score	Training Time (s)	Test Time (s)
**Multi-Sensor IoT Library Indoor Environment**	MCIMS	0.901	0.894	0.897	21.84	0.014
	KNN	0.872	0.865	0.868	8.63	0.032
	SVM	0.914	0.908	0.911	14.76	0.021
	Random Forest	0.931	0.926	0.928	19.42	0.018
	Decision Tree	0.887	0.881	0.884	6.94	0.009
	XGBoost	0.948	0.943	0.945	23.67	0.012
	Naive Bayes	0.821	0.814	0.817	4.28	0.008
	Logistic Regression	0.856	0.849	0.852	5.73	0.007
	LightGBM	0.953	0.949	0.951	18.35	0.011
	CatBoost	0.961	0.957	0.959	24.58	0.013
	ELM	0.903	0.897	0.900	7.82	0.009
	GPR	0.915	0.908	0.911	31.46	0.026
	DBN	0.939	0.933	0.936	28.73	0.019
	CPA-RAdaBoost [Proposed]	0.979	0.979	0.979	17.12	0.010

The conventional approaches pose considerable limitations in terms of real-time adaptive control in libraries. MCIMS [29] does not work well with dynamic environmental data since the latter changes rapidly over time. The KNN algorithm [30] uses lots of memory and performs slowly when inferring with the diverse sensor streams. The SVM [30] approach works under the presumption of the existence of linear separability and fails to take into account the nonlinear dependencies between the multi-sensor data. The DTs [30] are prone to overfitting and fail to generalize well to the changes in the indoor environment; although RF [30] improves the ensemble performance, it remains unable to capture the time-related dependencies that are crucial for adaptive control. XGBoost [30] is a resource-intensive method and therefore unsuitable for low-latency operations. The Naive Bayes [30] algorithm fails in cases involving interconnected environmental factors like temperature, humidity, and CO2 levels. Ultimately, Logistic Regression [30] is constrained by linear decision boundaries and does not effectively capture the intricate environmental dependencies required for controlling HVAC, lighting, and ventilation. The developed CPA-RAdaBoost scheme allows for intelligent regulation of the library environment through dynamic, real-time, and context-sensitive control while overcoming the drawbacks posed by traditional schemes. The introduced Multi-Sensor Internet of Things and AI-Driven Adaptive Management scheme incorporates multiple pieces of environmental information in a systematic way using preprocessing and feature refinement based on PCA to facilitate an accurate recognition of real-time indoor situations. Through the fusion of Random Forest, AdaBoost, and the CPA, this scheme can better handle feature selection, complex inter-relationships between environmental variables, and classification efficiency compared to existing machine learning algorithms. The identified environmental patterns are then used in adaptive closed-loop control of HVAC, lighting, and air circulation systems, providing excellent control accuracy (97.1%) with decreased response time (49 ms).

### Discussion

These findings show that the CPA-RAdaBoost model has successfully combined PCA, Random Forest, AdaBoost, and CPA to attain accurate environmental state recognition in minimal time periods while achieving better performance control. The model offers more effective adaptive control than conventional machine learning algorithms in capturing non-linear relationships between sensors and making control decisions according to the real-time environmental state. Energy prediction through IoT uses blockchain technology along with forecasting through the LSTM algorithm and EO-based hyperparameters, as proposed in the research [34]. Positive results in terms of forecasting and efficiency have been reported, but the effect of window size on accuracy and uncertainty has been noted. The technique applies only to predicting energy usage in smart home settings, not environmental monitoring scenarios.

Practically, the model will enable intelligent control of the HVAC, lighting, and ventilation systems through enhancing the occupant’s comfort while minimizing energy waste. The model may have been developed using library data, but it is still general enough to be applied in other indoor settings such as classrooms, hospitals, office buildings, museums, and commercial buildings, among others.

There are multiple limitations associated with this study. First, the framework has been tested against a single dataset available in the public domain and based on simulations rather than actual application. Second, issues such as the reliability of sensors, communication failure, and seasonality have not been addressed. In future studies, efforts will be made to test the framework in actual smart buildings and using large datasets.

## Conclusion

The major scientific novelty of the work lies in the creation of an integrated intelligent environmental management system that includes multi-sensor IoT sensing, ensemble learning optimization, and environment control all in one framework. In contrast to previous works that concentrate on separate tasks such as environmental monitoring, prediction, or energy optimization, the new framework considers all four key stages of environmental management: sensing/perception, state recognition using AI algorithms, learning through optimization, and control decision-making. The efficient Multi-sensor IoT and AI-based adaptive control systems of intelligent library indoor environments. The system delivers a very accurate recognition of environmental states and real-time closed-loop control through a heterogeneous Multi-Sensor IoT Library Indoor Environment Dataset and feature reduction through PCA using the optimized CPA-RAdaBoost ensemble model. All experiments and performances have been analyzed and evaluated with the use of Python based analytical tools and machine learning libraries. The obtained results include higher f1score of 0.987 for the temperature environment, recall of 0.940 for the ventilation environment, RMSE of 4.3 for dust, and average power consumption of 41.2W in the whole environment. The suggested framework also allows for scalability, easy integration into Building Management Systems, and occupant-aware energy efficiency, which makes it applicable in real-life smart buildings.

### Limitations and future scope

The proposed approach involves a data-driven control approach based on rules and does not consider any building thermal dynamics, airflows, or dynamics of the HVAC system. The combination of physical models of buildings or MPC with the proposed AI-based approach is planned for future research to enhance control performance.

The suggested model was tested on only one library database and simulations via software, which can affect the generalization capabilities of the framework in varied environmental and operating situations. The implementation of the framework in practical applications is expected to be affected by problems of sensor malfunctioning, network availability, and physical limitations. The future work will involve validation of the framework in varied smart buildings such as libraries, classrooms, hospitals, and offices on large-scale real-time Internet-of-Things data sets. PCA works well in reducing the number of dimensions and complexity; it integrates several environmental factors into principal components, making it difficult to physically interpret the contribution of each sensor. Despite this problem, PCA was employed to facilitate effective analysis of the heterogeneous sensor data from IoT and retain the most significant environmental data needed for precise adaptive control.

Though there are several indoor areas, various occupancy patterns, and a wide range of environmental parameters within the dataset, the dataset is that of a particular academic library only. In this way, any variation due to differences in architecture, HVAC system design, occupancy pattern, or climate could affect the applicability of the proposed model. The future scope of research would be to validate the proposed model by employing datasets from different libraries of varying climatic zones.

The proposed framework was validated using a single publicly available multi-sensor library indoor environment dataset, which may limit its generalizability across different building types and climatic conditions. The structured data set comprising time-stamped continuous observations constitutes a statistically sound and sufficient sample size to effectively capture microclimate transitions and assess the performance of the model as a closed loop control system. Further improvements would entail scaling up the application to cover multiple seasons to make it adaptive to different external climatic conditions. Future research would involve validating the model with data sets from various libraries, from different climatic zones and from IoT deployments in real time.

## Supporting information

S1 File Graphical abstract. (DOCX)

## References

[1] Widyardini ST, Pratiwi KY, Setyobudi E. Smart library: Using AI technology to enhance visitors’ comfort and books preservation through temperature and humidity adjustment in libraries. Knowl Garden: Int J Libr Stud. 2024;2(2):42–62.

[2] Marzouk M, Atef M. Assessment of indoor air quality in academic buildings using IoT and deep learning. Sustainability. 2022;14(12):7015. doi: 10.3390/su14127015

[3] Hou H (Cynthia). Investigating the impact of spaces design on user satisfaction in university libraries: A case study. F. 2025;43(7/8):571–93. doi: 10.1108/f-01-2024-0010

[4] Yang B, Zhao Z, Guo M, Li Z. Investigating the impact of indoor environment quality on user satisfaction in public library: A data fusion approach. J Build Eng. 2025;113:113984. doi: 10.1016/j.jobe.2025.113984

[5] Thneibat M. Assessing the post-COVID interaction between indoor environmental quality and occupants within educational buildings: A structural equation modeling approach. Build Environ. 2024;255:111422. doi: 10.1016/j.buildenv.2024.111422

[6] Peng L, Wei W, Gong Y, Jia R. University library space renovation based on the user learning experience in two Wuhan Universities. Int J Environ Res Public Health. 2022;19(16):10395. doi: 10.3390/ijerph191610395 36012030 PMC9408791

[7] Ceres G, Shindler L, Mercuri F, Zammit U. Development and application of affordable microclimate and indoor air quality monitoring platforms for historic libraries in cultural heritage preservation. Journal of Cultural Heritage. 2024;70:203–12. doi: 10.1016/j.culher.2024.09.007

[8] Ameen A, Bahrami A, Elosua Ansa I. Assessment of thermal comfort and indoor air quality in library group study rooms. Buildings. 2023;13(5):1145. doi: 10.3390/buildings13051145

[9] Lin J, Mylly N, Hedekvist PO, Shen J. A context-driven reliability framework for lighting systems in public libraries under extreme and varying environmental conditions. J Reliab Sci Eng. 2025;1(2):024001. doi: 10.1088/3050-2454/add162

[10] Hassan A, Zeeshan M, Bhatti MF. Indoor and outdoor microbiological air quality in naturally and mechanically ventilated university libraries. Atmospheric Pollut Res. 2021;12(8):101136. doi: 10.1016/j.apr.2021.101136

[11] Sahu V, Gurjar BR. Spatio-temporal variations of indoor air quality in a university library. Int J Environ Health Res. 2021;31(5):475–90. doi: 10.1080/09603123.2019.1668916 31547676

[12] Santos TLD, Cruz MNDSDL, Gioda A, Siqueira DYDS. Characterization of indoor air quality in a university library: Implications associated with pollutant emissions from new and old books and chemicals. Environ Forensics. 2024;26(4):512–28. doi: 10.1080/15275922.2024.2432495

[13] Opoku D-GJ, Perera S, Osei-Kyei R, Rashidi M, Bamdad K, Famakinwa T. Digital twin for indoor condition monitoring in living labs: University library case study. Automat Construc. 2024;157:105188. doi: 10.1016/j.autcon.2023.105188

[14] Song G, Ai Z, Zhang G, Peng Y, Wang W, Yan Y. Using machine learning algorithms to multidimensional analysis of subjective thermal comfort in a library. Build Environ. 2022;212:108790. doi: 10.1016/j.buildenv.2022.108790

[15] Zhang D, Mui K-W, Masullo M, Wong L-T. Application of machine learning techniques for predicting students’ acoustic evaluation in a university library. Acoustics. 2024;6(3):681–97. doi: 10.3390/acoustics6030037

[16] Aflaki A, Esfandiari M, Jarrahi A. Multi-criteria evaluation of a library’s indoor environmental quality in the tropics. Buildings. 2023;13(5):1233. doi: 10.3390/buildings13051233

[17] Chang L, Permana I, Setiawan T, Agharid AP, Wang F. Evaluating and enhancing thermal comfort and indoor air quality in a library building through measurement and simulation. Ain Shams Eng J. 2025;16(10):103580. doi: 10.1016/j.asej.2025.103580

[18] Xu S, Chen Y, Liu J, Kang J, Gao J, Qin Y, et al. Comprehensive improvement of energy efficiency and indoor environmental quality for university library atrium—A multi-objective fast optimization framework. Front Arch Res. 2025;14(2):449–70. doi: 10.1016/j.foar.2024.08.010

[19] Godasiaei SH, Ejohwomu OA, Zhong H, Booker D. Integrating experimental analysis and machine learning for enhancing energy efficiency and indoor air quality in educational buildings. Build Environ. 2025;276:112874. doi: 10.1016/j.buildenv.2025.112874

[20] Zou Y, Zou W, Chen H, Dong X, Zhu L, Shu H. Research on real-time control strategy for HVAC systems in university libraries. Appl Sci. 2025;15(5):2855. doi: 10.3390/app15052855

[21] Liu X, Chen X, Zhang J, Liu L. Optimizing HVAC performance through occupancy-based control: A machine learning approach for energy efficiency and comfort. Energy Build. 2025;347:116326. doi: 10.1016/j.enbuild.2025.116326

[22] Xie H, Li J. Study on indoor ventilation and comfort of university libraries in hot and dry areas in summer-taking a university in Xinjiang. Thermal Sci Eng Progress. 2025;63:103706. doi: 10.1016/j.tsep.2025.103706

[23] Djabir Abdoulaye D, Mohamad Nur Hidayat M, Azian H. CFD Investigation on Thermal Comfort in Open Spaces Library in Tropical Climate. CFD Lett. 2023;15(9):83–101. doi: 10.37934/cfdl.15.9.83101

[24] Shi K, Ren J, Cao X, Kong X. Optimizing thermal comfort in an atrium-structure library: On-site measurement and TRNSYS-CONTAM co-simulation. Build Environ. 2024;266:112041. doi: 10.1016/j.buildenv.2024.112041

[25] Haiying W, Fengming Z, Jiankai L, Hang M, Huxiang L. Effects of different zoning thermostat controls on thermal comfort and cooling energy consumption in reading rooms of a library. Energy. 2024;292:130507. doi: 10.1016/j.energy.2024.130507

[26] Li Y, He J. Evaluating the improvement effect of low-energy strategies on the summer indoor thermal environment and cooling energy consumption in a library building: A case study in a hot-humid and less-windy city of China. Build Simul. 2021;14(5):1423–37. doi: 10.1007/s12273-020-0747-6

[27] Rosa-Bilbao J, Butt FS, Merkl D, Wagner MF, Schäfer J, Boubeta-Puig J. IoT-based indoor air quality management system for intelligent education environments. IEEE Internet Things J. 2025;12(11):18031–41. doi: 10.1109/jiot.2025.3539886

[28] Rastogi K, Lohani D. Context-aware IoT-enabled framework to analyse and predict indoor air quality. Intell Syst Appl. 2022;16:200132. doi: 10.1016/j.iswa.2022.200132

[29] Bakare MS, Abubaker K. IoT-based indoor environmental monitoring system using multi-parameter sensing and ESP32-WROOM integration. Discov Electron. 2026;3(1). doi: 10.1007/s44291-026-00157-3

[30] Mammadov S, Kucukkulahli E. A user-centric smart library system: IoT-Driven environmental monitoring and ML-based optimization with future Fog–cloud architecture. Appl Sci. 2025;15(7):3792. doi: 10.3390/app15073792

[31] Mallala B, Ahmed AIU, Pamidi SV, Faruque MO, Reddy R. Forecasting global sustainable energy from renewable sources using random forest algorithm. Result Eng. 2025;25:103789. doi: 10.1016/j.rineng.2024.103789

[32] Zhao J, Li D, Zhou J, Armaghani DJ, Zhou A. Performance evaluation of rock fragmentation prediction based on RF‐BOA, AdaBoost‐BOA, GBoost‐BOA, and ERT‐BOA hybrid models. Deep Underground Sci Eng. 2024;4(1):3–17. doi: 10.1002/dug2.12089

[33] He X, Shan W, Zhang R, Heidari AA, Chen H, Zhang Y. Improved colony predation algorithm optimized convolutional neural networks for electrocardiogram signal classification. Biomimetics (Basel). 2023;8(3):268. doi: 10.3390/biomimetics8030268 37504156 PMC10377160

[34] Pawar PP, Kumar D, Addula SR, Cheema QN, Haq AU, Meesala MK. A blockchain-based IoT framework for smart homes: Enhancing energy prediction and security with LSTM and equilibrium optimization. In: 2025 International Conference on Intelligent and Cloud Computing (ICoICC). IEEE; 2025. pp. 1–8. doi: 10.1109/ICoICC64033.2025.11052010

