# Supplementary material for: Multi-sensor IoT data and AI-driven adaptive control model for library indoor environments
Source: PLoS One. 2026 Oct 27;21(10):e0361038. doi: 10.1371/journal.pone.0361038 (PMC13712579; doi:10.1371/journal.pone.0361038)
Supplement: Supplementary file 1 [file pone.0361038.s001.docx]

*Graphical abstract*
